# Genomic sequence capture of *Plasmodium relictum* in experimentally infected birds

**DOI:** 10.1186/s13071-022-05373-w

**Published:** 2022-07-29

**Authors:** Vincenzo A. Ellis, Victor Kalbskopf, Arif Ciloglu, Mélanie Duc, Xi Huang, Abdullah Inci, Staffan Bensch, Olof Hellgren, Vaidas Palinauskas

**Affiliations:** 1 Molecular Ecology and Evolution Laboratory, Department of Biology, Lund University, S-22362 Lund, Sweden; 2 Department of Entomology and Wildlife Ecology, University of Delaware, Newark, DE USA; 3 Department of Parasitology, Faculty of Veterinary Medicine, Erciyes University, 38280 Kayseri, Turkey; 4 Vectors and Vector-Borne Diseases Implementation and Research Center, Erciyes University, 38280 Kayseri, Turkey; 5 Nature Research Centre, Akademijos 2, 08412 Vilnius, Lithuania; 6 MOE Key Laboratory for Biodiversity Science and Ecological Engineering, College of Life Sciences, Beijing Normal University, Beijing, People’s Republic of China

**Keywords:** Avian malaria, Parasitemia, Haemosporida, Hybrid enrichment, Parasite genomics

## Abstract

**Background:**

Sequencing parasite genomes in the presence of host DNA is challenging. Sequence capture can overcome this problem by using RNA probes that hybridize with the parasite DNA and then are removed from solution, thus isolating the parasite DNA for efficient sequencing.

**Methods:**

Here we describe a set of sequence capture probes designed to target 1035 genes (c. 2.5 Mbp) of the globally distributed avian haemosporidian parasite, *Plasmodium relictum*. Previous sequence capture studies of avian haemosporidians from the genus *Haemoproteus* have shown that sequencing success depends on parasitemia, with low-intensity, chronic infections (typical of most infected birds in the wild) often being difficult to sequence. We evaluate the relationship between parasitemia and sequencing success using birds experimentally infected with *P. relictum* and kept under laboratory conditions.

**Results:**

We confirm the dependence of sequencing success on parasitemia. Sequencing success was low for birds with low levels of parasitemia (< 1% infected red blood cells) and high for birds with higher levels of parasitemia. *Plasmodium relictum* is composed of multiple lineages defined by their mitochondrial DNA haplotype including three that are widespread (SGS1, GRW11, and GRW4); the probes successfully isolated DNA from all three. Furthermore, we used data from 25 genes to describe both among- and within-lineage genetic variation. For example, two samples of SGS1 isolated from different host species differed by 11 substitutions across those 25 genes.

**Conclusions:**

The sequence capture approach we describe will allow for the generation of genomic data that will contribute to our understanding of the population genetic structure and evolutionary history of *P. relictum*, an extreme host generalist and widespread parasite.

**Graphical Abstract:**

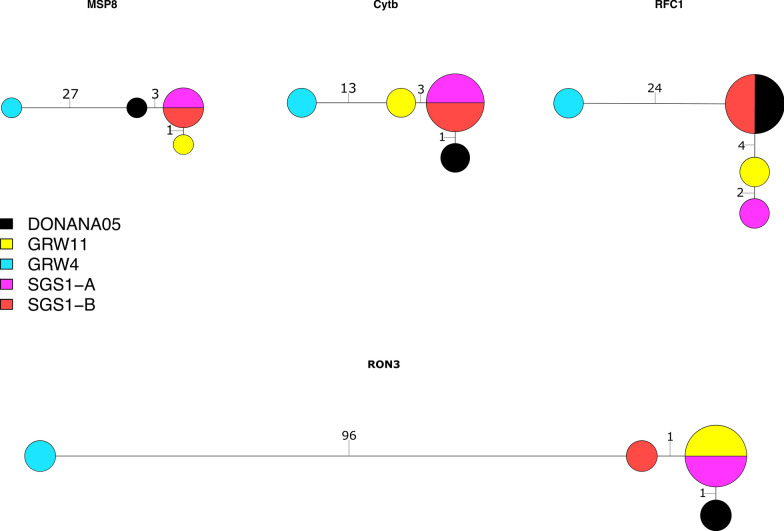

**Supplementary Information:**

The online version contains supplementary material available at 10.1186/s13071-022-05373-w.

## Background

Direct sequencing of intracellular parasite DNA can be challenging as host DNA is often much more abundant than parasite DNA in a biological sample [1, 2]. Nevertheless, genome-level analyses of parasites have become relatively common, partly because deep sequencing has become cheaper and partly as a result of the development of DNA enrichment techniques [3]. Such techniques include sequencing parasite transcriptomes [4], selective whole genome amplification [5], long-range polymerase chain reaction (PCR) [6, 7], and sequence capture (also known as hybrid enrichment [8]). Sequence capture is particularly promising as a parasite DNA enrichment technique because it can be applied to previously collected and even degraded DNA samples. For example, Marciniak et al. [9] used sequence capture to sequence approximately half of the mitochondrial genome of the human malaria parasite *Plasmodium falciparum* in human molars from the first to second centuries CE.

Sequence capture works by designing biotinylated RNA probes that are complementary to parts or all of a genome of interest. The probes are incubated with DNA (both target and non-target DNA) that has been extracted from a biological sample to promote hybridization with the target DNA. Probes bound to the target DNA then bind to streptavidin-coated magnetic beads that are added to solution. The beads are fixed in place with a magnet as the rest of the solution is removed by pipetting and the target DNA is isolated. A sequencing library is built from the target DNA and sequenced on a high-throughput sequencing platform. While sequence capture can facilitate the sequencing of parasite genomes in previously collected samples, rigorous validation of sequence capture protocols is necessary to understand their effectiveness with different kinds of samples. For example, sequence capture may be ineffective if the amount of parasite DNA in a sample falls below a certain threshold [10].

Avian haemosporidian parasites (phylum: Apicomplexa, order: Haemosporida) of the genera *Plasmodium*, *Haemoproteus*, and *Leucocytozoon* are common, diverse parasites of birds found on all continents except Antarctica [11, 12]. Their life-cycles are complex and include two hosts, a dipteran vector and an avian host. They reproduce sexually in their dipteran vectors and are transmitted to avian hosts when the vector takes a blood meal. Once the parasite is inside its avian host, it enters the host’s solid tissues (i.e., hepatocytes, reticular cells or endothelial cells of different organs) and eventually infects the host’s red blood cells. Once in the red blood cells, some parasite cells (gametocytes) can be transmitted to the next competent vector that feeds on the avian host [12]. Most research into avian haemosporidians has been conducted on parasites found in bird blood through microscopy [12] and molecular methods [13]. Haemosporidian mitochondrial genes are readily amplified by PCR and have been used for barcoding and detecting parasites [13, 14]. However, avian red blood cells are nucleated, making it inevitable that a sample of avian blood will contain much more host DNA than parasite DNA. This in turn makes direct sequencing of the parasite genome from a sample of bird blood nearly impossible [15]. Several approaches have emerged to solve the problem of sequencing avian haemosporidian genomes, including harvesting the parasite from dipteran hosts [16], transcriptome sequencing [17–22], and causing the exit of parasites from red blood cells and exflagellation after exposing infected blood to air [1, 2].

Once the first avian haemosporidian genomes and transcriptomes became available, it was possible to design sequence capture protocols to separate parasite DNA from host DNA in previously collected samples [10, 23]. Huang et al. [23] designed sequence capture probes to target 1000 genes (c. 2.29 Mbp) from the genome of *Haemoproteus tartakovskyi*. The authors tested those probes in multiple parasites and found that sequencing success was negatively related to the phylogenetic distance of the parasite being tested relative to the reference genome. Barrow et al. [10] used the sequencing results of three parasite lineages from Huang et al. [23] to design a set of sequence capture probes that would better capture a phylogenetically diverse set of parasites. The authors tested their protocol on DNA extracted from the blood of naturally infected birds and demonstrated a positive relationship between parasitemia (the proportion of host erythrocytes infected by the parasite) and sequencing success. The authors were able to sequence many parasite loci from many diverse parasites and used those genetic data to construct robust phylogenies [10].

Given the successful application of sequence capture to parasites in the genus *Haemoproteus* [10, 23], we set out to design and validate a sequence capture protocol for an avian *Plasmodium* parasite. We designed probes that target 1035 genes (c. 2.5 Mbp) of the genome of *Plasmodium relictum* [16] that would allow us to improve the genomic resolution of previous phylogenetic and phylogeographic studies of the parasite [24]. *Plasmodium relictum* is a widely distributed parasite composed of three common, well-studied mitochondrial lineages (SGS1, GRW11, and GRW4; the clade also includes many other lineages [25]) that have been identified by sequence divergence in part of their mitochondrial cytochrome *b* (cyt *b*) genes [24]. At least one of these lineages (GRW4) has been implicated as the causative agent in the population declines of many native birds in Hawaii [26, 27]. The three mentioned lineages of *P. relictum* are extreme host generalists relative to most avian haemosporidian parasites [25], making them particularly interesting from the perspective of understanding the genetic underpinnings of host specificity [16]. Hellgren et al. [24] assembled samples of *P. relictum* from around the world and amplified a section of a nuclear gene of the parasite (merozoite surface protein 1, MSP1) to perform phylogeographic analyses and reconstruct the historical biogeography of the parasite. They identified two MSP1 haplotypes in the GRW4 lineage that are present in North and South America and in Hawaii, suggesting that Hawaii was colonized by at least two strains that dispersed to Hawaii from North and/or South America (likely with human assistance [26, 28]). Furthermore, Hellgren et al. [24] determined that SGS1 and GRW11 shared MSP1 haplotypes, suggesting that those two lineages are not reproductively isolated. Further genomic data generated by sequence capture from previously collected samples of *P. relictum* will likely bring greater resolution to many of the phylogeographic and phylogenetic patterns that have been assessed previously with single genetic markers [24, 29].

Here we present the sequence capture probes and protocol that we have used to sequence *P. relictum* DNA from samples of infected avian blood. We evaluated the efficacy of the protocol in relation to parasitemia using blood samples collected from experimentally infected, captive birds. Infecting birds with a single parasite lineage in a controlled setting allowed us to avoid mixed infections influencing our results. We test whether these probes work on all three common *P. relictum* lineages and investigate whether the targeted genes reveal novel relationships among multiple *P. relictum* isolates.

## Methods

### Sequence capture design

We designed sequence capture probes to match parts of the genome of *P. relictum* sequenced by Böhme et al. [16]. This genome sequence is from the lineage DONANA05 which differs from SGS1 by one nucleotide at the mitochondrial gene cyt *b*. We selected genes that are known to have orthologous copies across 17 apicomplexan parasite species [1], genes with known associations to cell invasion biology, mitochondrial genes, and genes that were picked at random from the published genome of *P. relictum* [16]. All mapping and analyses of our sequence data use version 54 of the reference genome (plasmodb.org), but initial probe selection used an earlier version. We created a FASTA file of regions to sequence and sent this file to Agilent Technologies, which used proprietary software to remove sequences that were likely to match equally well to avian DNA, making the final sequences more parasite-specific. They then produced a list of 120-bp-long probes (Additional file 1: File S1) that covered each nucleotide in our selected sequences once (i.e., 1× tiling). The probes cover all or parts of 1035 genes in the version 54 of the reference genome, including two mitochondrial genes, cyt *b* and cytochrome c oxidase subunit 1 (COX1; Additional file 4: Table S1). The third protein coding mitochondrial gene (cytochrome c oxidase subunit 3) was inadvertently left out of our list of targeted regions but can be easily added into future versions of this probe-set. Finally, Agilent Technologies produced a sequence capture kit (SureSelect^XT^ Target Enrichment System for Illumina paired-end multiplexed sequencing) which we used in this study.

### Experimental infections and parasitemia

The samples were obtained from previous experimental studies carried out in 2011, 2013 and 2017, all confirmed to be infections of single mitochondrial lineages (Additional file 5: Table S2). Sixteen samples (lineage SGS1) were obtained from experimentally infected siskins (*Spinus spinus*). Those birds were originally infected with an isolate from a red crossbill (*Loxia curvirostra*) and multiplied in the siskins in 2013. Another SGS1 isolate was obtained from a house sparrow (*Passer* *domesticus*) and multiplied in canaries (*Serinus canaria*); two samples (735 and 739) from infected canary blood were used for this analysis. To distinguish the two SGS1 isolates, we refer to SGS1 isolated from a red crossbill as SGS1-A and SGS1 isolated from a house sparrow as SGS1-B. A blood sample with the *P. relictum* GRW11 lineage was obtained from an experimentally infected crossbill and that lineage was originally isolated from a house sparrow in 2011. All of these lineages were isolated from birds caught at the Biological Station of the Zoological Institute of the Russian Academy of Sciences on the Curonian Spit in the Baltic Sea (55°05′N, 20°44′E) and confirmed to represent single (i.e., not mixed) infections. Parasitemia was determined by microscopy at several points during infection and is represented as a percentage by counting the number of infected red blood cells per 1000 or 10,000 erythrocytes [30]. More detailed information about each sample can be found in Additional file 5: Table S2. The GRW4-infected bird was a juvenile great reed warbler (*Acrocephalus arundinaceus*) from Bulgaria that was experimentally infected with GRW4 as part of another study [31].

Infections were initially identified using a standard nested PCR reaction targeting part of the mitochondrial cyt *b* gene [14, 32] and Sanger sequencing as reported in Hellgren et al. [24].

### Sequence capture protocol and sequencing

We used c. 200 ng of DNA isolated from the blood of infected birds for sequence capture following the approach detailed in Huang et al. [23]. DNA was quantified using a Qubit 2.0 Fluorometer (Thermo Scientific, Waltham, MA, USA). We sheared the DNA to c. 300-bp fragments using a M220™ Focused-ultrasonicator (Covaris, MA, USA) and then proceeded to perform the sequence capture protocol outlined by Agilent Technologies for the SureSelect^XT^ kit. We incubated the probes with samples for 24 h and we used 18 cycles of PCR in the post-capture amplification with indexing primers (instead of the recommended 10 to 16 cycles) to ensure sufficient DNA for sequencing. Captured samples were sequenced on an Illumina MiSeq instrument at the Lund University DNA Sequencing Facility. The samples in this study were sequenced in two runs. The GRW11 and GRW4 samples along with one of the SGS1 samples were sequenced in one run and the rest of the SGS1 samples were sequenced in another (Additional file 5: Table S2).

### Bioinformatics analyses

We processed the raw sequencing reads using Trimmomatic v0.39 [33] by removing adapter sequences (adapter detection settings: seed mismatched = 2, palindromic clip threshold = 30, simple clip threshold = 10, min adapter length = 8, keep both reads = TRUE) and removing low-quality reads with a sliding window (window size = 4, required quality = 15). Read quality was assessed with FastQC v0.11.9 [34]. The reads were mapped to the *P. relictum* genome (plasmodb.org, version 54) using Nextgenmap v0.5.5 [35] with default options. The resulting sam files were compressed to BAM files, sorted and indexed using SAMtools v1.15.1 [36]. MarkDuplicates v2.20.8 in Picard tools [37] was used to identify duplicate reads. Success of sequence capture was measured in several ways. We calculated the percentage of the targeted *P. relictum* nucleotides that were sequenced to a depth of coverage of 5×, 20×, 50×, and 100× for each sample using Qualimap BamQC [38]. Qualimap was provided with the coordinates of the targeted exons. We further calculated the number of mapped and unmapped reads using the stats program in SAMtools. MultiQC [39] was used to summarize results from FastQC, Qualimap, and SAMtools stats. Unmapped reads were BLASTed to check for identity. Keanu [40] was used to organize the BLAST hits into taxonomic categories.

### Statistical analyses

We compared the measures of sequence capture success with parasitemia determined by microscopy using Spearman’s non-parametric correlation tests. Graphics were produced using the ggplot2 R package [41] and all statistical analyses were conducted with R v4.1.1 [42].

### Coverage analysis

To visualize the evenness of coverage/depth of the reads across the genome, depth of coverage information was extracted from the BAM files using bedtools v2.30.0 [43] *genomecov* and exported as bedgraph files. Bedtools counts the number and length of reads across the entire chromosome. The bedgraph files were imported into R, the short archived contigs were filtered out (leaving the “non-archived chromosomes”, i.e., chromosomes 1–14 and the apicoplast and mitochondrial genomes), and the absolute coverage (i.e., number of reads mapped to each nucleotide in the chromosome) was plotted for each chromosome using ggplot2. A 17 kb region of chromosome 1 was identified for comparison of coverage across three samples that represent low (sample 1455, 0.1%), medium (1313, 7%), and high parasitemia (1459, 56.4%; Additional file 5: Table S2); difference in depth of coverage was visualized in the Integrative Genomics Viewer (IGV) [44].

A few randomly selected regions of extremely high coverage (i.e., > 1000× in a high-coverage/parasitemia sample, sample 1309, and > 250× in a low-coverage/parasitemia sample, sample 1457, referred to as “spikes”) that were targeted by the probes and regions that were not targeted by the initial probe-set but still had reads mapped to a depth of coverage of at least 20× were extracted with bedtools *coverage,* awk, and bedtools *getfasta* for further analysis. This analysis included BLAST [45] and visual inspection of the sequences in order to understand the cause of the spikes and non-target mapping.

We also conducted a rarefaction analysis of sequence coverage. Specifically, we investigated the relationship between the number of probed regions intersected by a sequenced read (by at least 10 bp) and number of sequenced reads that mapped to a probed region for each sample. We were interested in using this analysis to understand whether samples had been sequenced to saturation and if low parasitemia samples could gain complete coverage through additional sequencing. We counted the number of overlapping reads using the program featureCounts [46] and the vegan v2.5-7 R package [47] was used to construct the curves. We similarly determined the number of reads intersecting each targeted gene and each probe by at least 10 bp for each sample to characterize sequence coverage across the samples.

Several probes targeted highly repetitive regions in the reference genome (as determined with the program D2RReadFilter [48] using default options) and/or resulted in uninformative spikes in depth of coverage (we used a threshold of 1000 in the high coverage sample and 250 in the low coverage sample to identify the spikes, excluding probes that targeted the mitochondrial genes as those genes typically had high coverage across samples). We identified those probes from a high-parasitemia sample (1309) and low-parasitemia sample (1457) and present them in Additional file 4: Table S1 so that they can be removed in future designs.

### Haplotype networks

We aligned the sequences from each of 25 genes (Additional file 6: Table S3) from four samples in our study (1309, lineage SGS1-A originally isolated from a red crossbill; 51242, lineage GRW4 originally isolated from a great reed warbler; 735, lineage SGS1-B originally isolated from a house sparrow; cc82, lineage GRW11 originally isolated from a house sparrow) to the reference genome sequence, i.e., the lineage DONANA05. The genes were chosen haphazardly, but we purposely included several parasite surface protein genes that are thought to interact with host cells (e.g., rhoptry neck proteins 3 [RON3] and 12 [RON12]) and the two mitochondrial genes targeted by the probe set. We produced the alignments using MUSCLE v3.8.31 [49]. We constructed haplotype networks for four genes (cyt *b*, MSP8, replication factor C subunit 1, and RON3) to illustrate various relationships among the samples. We produced the haplotype networks with R packages ape v5.5 [50], pegas v1.0-1 [51], reshape v0.8.8 [52], and geiger v2.0.7 [53]. We also concatenated the 25 genes and calculated the nucleotide differences between the lineages and isolates using ape.

## Results

### General sequence characteristics

We sequenced an average of 2,988,012 ± 3,150,933s.d. reads from the samples, with 1,487,563 ± 1,436,092 mapping to the reference genome and 1,500,449 ± 3,150,550 not mapping. None of the unmapped reads BLASTed to *Plasmodium* (Table 1).

**Table 1:** BLAST results of unmapped reads against the refseq database for five samples representing the three lineages in this study (GRW11, GRW4, SGS1) and the two SGS1 lineage isolates (SGS1-A and SGS1-B)

BLAST Taxon Hit	GRW11 (cc82)	GRW4 (51242)	SGS1-A (1309)	SGS1-A (1455)	SGS1-B (735)
Aves: Passeriformes	6342	39,851	2258	14,009	1900
Aves: Non-passerine orders	322	3734	89	910	90
Mammalia	10	150	3	31	4
*Plasmodium*	0	0	0	0	0
All hits	16,478	82,689	9782	30,536	5467
Total unmapped reads	991,173	14,761,081	360,028	1,593,536	423,348
No BLAST hit	974,695	14,678,392	350,246	1,563,000	417,881
All reads	6,116,400	15,219,066	5,915,344	1,839,486	1,939,288

Two samples from the SGS1-A lineage isolate were BLASTed; they represent a high-parasitemia (1309) and low-parasitemia (1455) sample. The number of reads that mapped to birds (shown separately for Passeriformes and non-passerine orders), mammals (Mammalia), and Plasmodium (no unmapped reads mapped to Plasmodium) are shown. The “All hits” category is the sum of the reads in the aforementioned categories and all other unmapped reads that resulted in hits to other taxonomic categories (including unassigned taxa; data not shown). The number of unmapped reads that did not result in a BLAST hit (“No BLAST hit”), the total number of unmapped reads for each sample (“Total unmapped reads”), and the total number of sequenced reads for each sample (mapped and unmapped; “All reads”) are also shown. Sample name follows lineage name in parentheses

### Experimental infections of SGS1

**Fig. 1:**
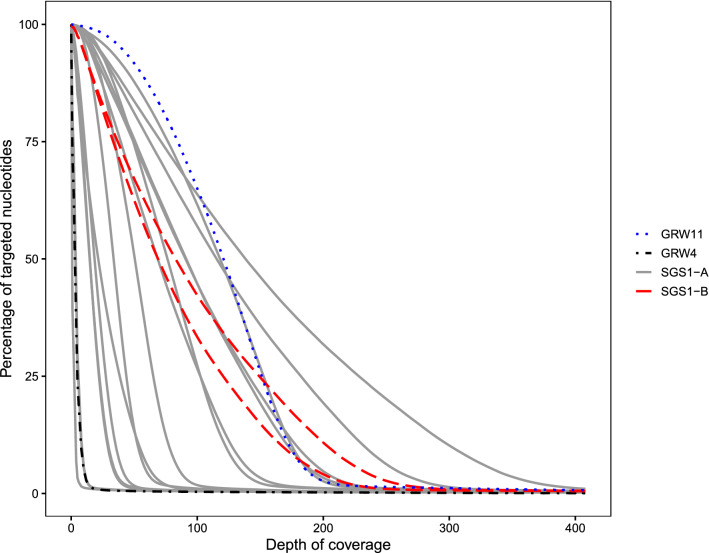
The percentage of targeted nucleotides as a function of depth of coverage at which those nucleotides were sequenced. Steeply sloping lines on the left-hand side of the graph suggest poor sequencing relative to lines with shallower slopes closer to the right-hand side of the graph. Each line represents a single sample and line type and color correspond to the lineage isolates. The lineage SGS1 was represented by two isolates; SGS1-A was originally isolated from the host species *Loxia curvirostra* and SGS1-B from *Passer* *domesticus*

**Fig. 2:**
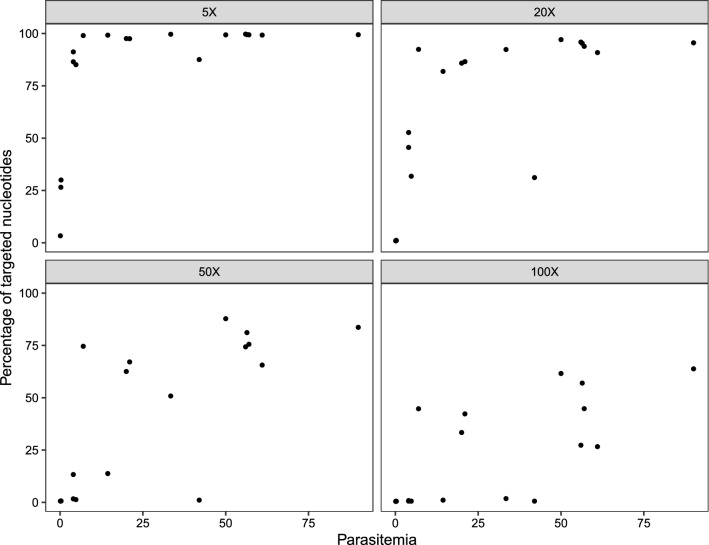
The percentage of nucleotides that the sequence capture probes were designed to capture sequenced at different depths of coverage (5×, 20×, 50×, 100×) in relation parasitemia (percentage of infected red blood cells). Samples are from experimental infections of the lineage SGS1

The depth of coverage varied among samples (Fig. 1). Among the experimental infections of SGS1, variation in sequencing success was dependent on parasitemia. Parasitemia was positively correlated with the percentage of targeted nucleotides that were sequenced to a depth of coverage of 5× (*ρ* = 0.835, *P* < 0.001), 20× (*ρ* = 0.803, *P* < 0.001), 50× (*ρ* = 0.795, *P* < 0.001), and 100× (*ρ* = 0.785, *P* < 0.001; Fig. 2). Sequence capture from the experimental infection of SGS1 with the lowest parasitemia (0.1%) resulted in only 3.32% of the targeted nucleotides from the *P. relictum* genome being sequenced to a depth of coverage of 5× (Additional file 5: Table S2). However, sequencing success increased nonlinearly with parasitemia (Fig. 2) and the next highest parasitemia in an experimental infection (0.24%) resulted in 26.51% of targeted nucleotides being sequenced to a depth of coverage of 5× (parasitemia of 0.3% resulted in 29.97% of targeted nucleotides sequenced to 5×). The infection of SGS1 with the highest parasitemia (90%) resulted in 99.41% of the targeted nucleotides being sequenced to a depth of coverage of 5× (Additional file 5: Table S2).

The ratio of reads that mapped to the reference genome to reads that did not map to the reference genome in experimental infections of SGS1 were positively correlated with parasitemia (*ρ* = 0.814, *P* < 0.001).

### Experimental infections of GRW11 and GRW4

The infection of GRW11 was derived from an experimentally infected bird and reached a high parasitemia (8%) and sequencing success was high; 99.78% of the targeted nucleotides were sequenced to a depth of coverage of 5×. The GRW4 infection was also derived from an experimental infection, but only 23.91% of the targeted nucleotides were sequenced to a depth of coverage of 5×. This may have been a result of GRW4’s greater phylogenetic distance from SGS1 [23, 24] and lower parasitemia (< 1% [31]).

### Evenness of coverage

**Fig. 3:**
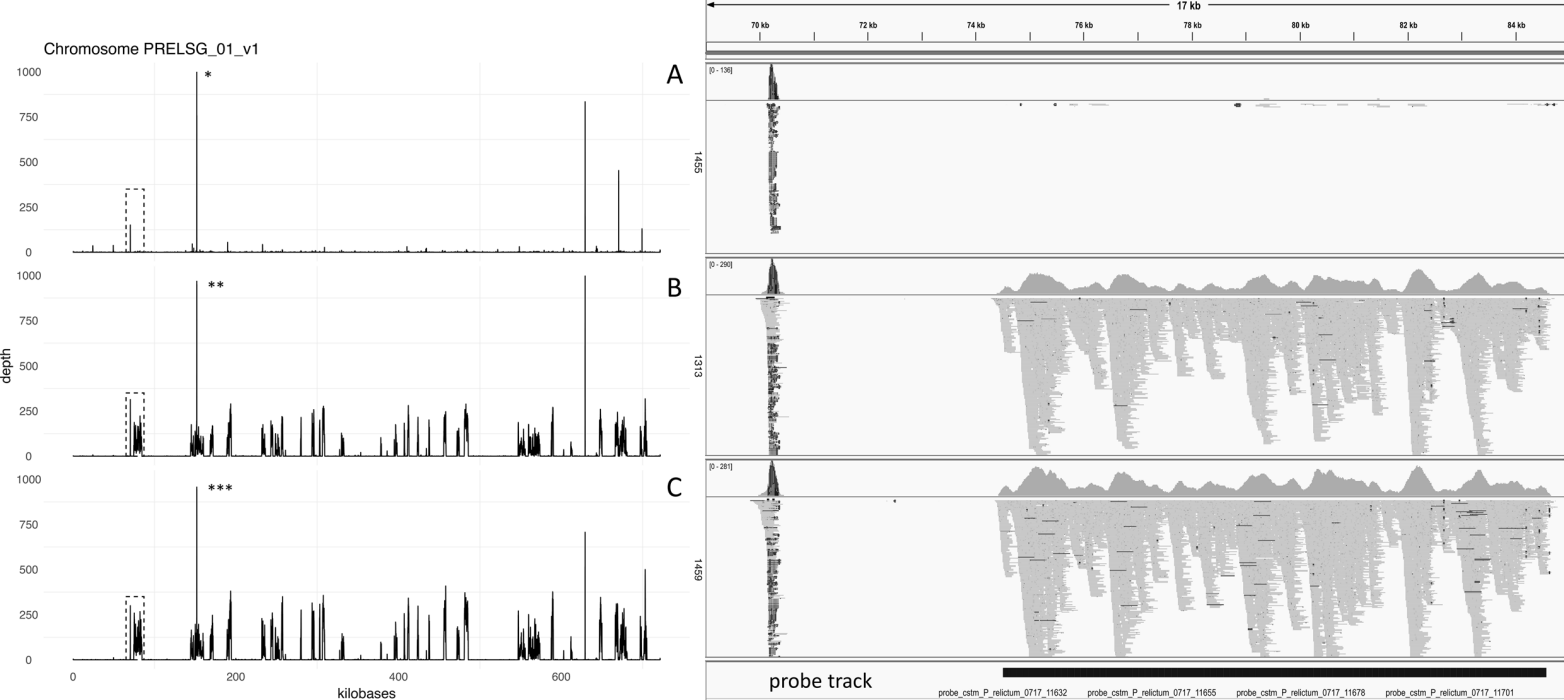
Examples of mapping depth over the first chromosome for low—0.1% (**a**), medium—7% (**b**), and high—56.4% (**c**) parasitemia samples with the entire chromosome on the left and a region taken from the dashed box expanded on the right in IGV. *Depth extends to 4015. **Depth extends to 1758. ***Depth extends to 2711

When plotting absolute depth of mapping across each chromosome, coverage is highly variable among samples (Additional file 1: Figure S1) and along chromosomes (Fig. 3). Spikes in coverage were typically short (< 300 bp) and occurred both in the probed regions and outside the probed regions (Fig. 3). It is not unusual to see spikes with depth of coverage over 2000 times higher than in neighboring regions in most samples and chromosomes. They tended to be of low complexity. When randomly selected sequences that represent these spikes were BLASTed, ribosomal genes were found (results not shown). This may suggest that the high coverage represents host reads which map to highly conserved ribosomal regions. We identified probes that targeted highly repetitive regions or regions that led to spikes in coverage from both a high (1309) and a low (1457) parasitemia sample so that those probes can be removed from future designs (Additional file 4: Table S1).

The effect of parasitemia on the breadth and depth of coverage (Fig. 2) can also be observed when visually examining the samples with low (0.1%), medium (7%), and high (56.4%) parasitemia (Fig. 3), where there is a large increase between 0.1 and 7%, but less of a difference between 7 and 56.4% parasitemia. In cases where we observed mapped reads outside probed regions, we found they tended to be of low complexity and highly repetitive (data not shown). Furthermore, full sequence coverage (i.e., reads mapping to all probed regions) for samples with parasitemia lower than 1% likely cannot be attained with more sequencing based on a comparison of rarefaction curves (Additional file 2: Figure S2). While one or more reads mapped to most targeted genes for all samples (Additional file 7: Table S4), coverage across genes (as indicated by the number of reads mapping to each probe) was low (Additional file 8: Table S5).

### Haplotype networks

**Fig. 4:**
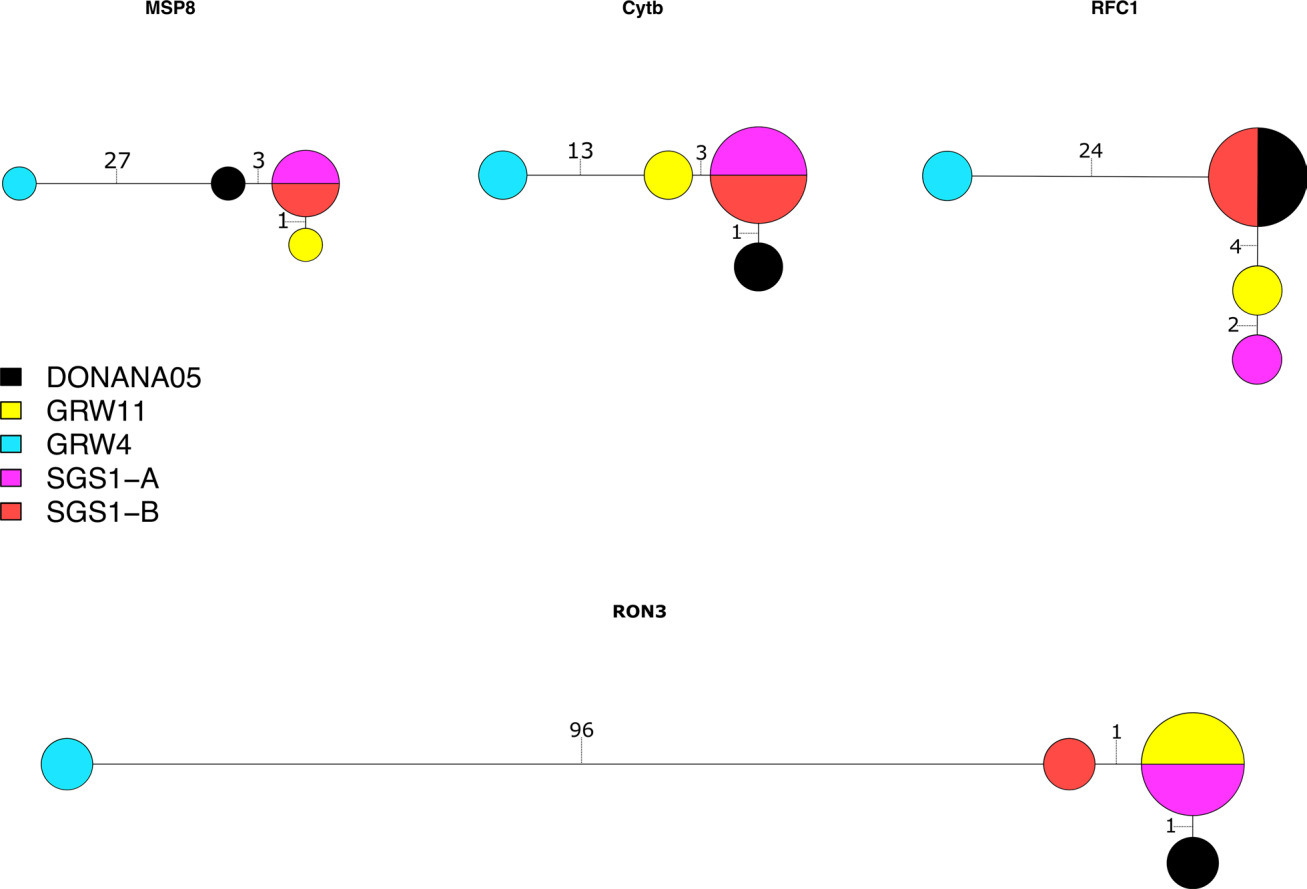
Haplotype networks of lineages (DONANA05 is the lineage name of the reference genome; GRW11 is the lineage of sample cc82; GRW4 is the lineage of sample 51242; SGS1-A is lineage SGS1 represented by sample 1309; SGS1-B is lineage SGS1 represented by sample 735) for four genes (merozoite surface protein 8, MSP8, 1321 bp; cytochrome *b*, cyt *b* 1151 bp; replication factor C subunit 1, RFC1 2380 bp; rhoptry neck protein 3, RON3, 4558 bp). The number of substitutions separating haplotypes is presented on each branch of the network

As previously shown, the lineages GRW11 and the reference sequence differ from SGS1 by one mutation in the barcoding region of the cyt *b* gene while GRW4 shows greater divergence from that group (Fig. 4). However, these relationships are different for other genes (Fig. 4). In fact, for at least two genes (replication factor C subunit 1 and RON3) samples 735 (lineage SGS1-B) and 1309 (lineage SGS1-A) do not share haplotypes. Samples 1309 and the reference share their replication factor C subunit 1 haplotypes while 1309 and cc82 (GRW11) share their RON3 haplotypes. Concatenating the 25 genes revealed many nucleotide differences between the lineages and 11 separating the two SGS1 samples that originated from different hosts (Table 2).

**Table 2:** A distance matrix of number of nucleotide differences between lineages (DONANA05 is the lineage name of the reference genome; GRW11 is the lineage of sample cc82; GRW4 is the lineage of sample 51242; SGS1-A is lineage SGS1 represented by sample 1309; SGS1-B is lineage SGS1 represented by sample 735) computed from the concatenated alignment of 25 genes (Additional file 6: Table S3)

	SGS1-A	SGS1-B	GRW11	DONANA05
SGS1-A				
SGS1-B	11			
GRW11	19	22		
DONANA05	16	9	23	
GRW4	666	663	675	660

## Discussion

Here we introduced a sequence capture probe-set and protocol for the cosmopolitan, generalist avian malaria parasite *P. relictum*. The protocol can capture DNA from three well studied and common *P. relictum* lineages: SGS1, GRW11, and GRW4. Sequence capture success follows a nonlinear relationship with parasitemia (Figs. 2 and 3). Therefore, even small differences in parasitemia can lead to large gains in sequencing success up to a threshold value of c. 4% parasitemia at which point most targeted nucleotides are sequenced to a depth of coverage of at least 5× (Fig. 2). We also demonstrate that many unmapped reads represent residual DNA from birds or other sources, but none of the unmapped reads are from *Plasmodium* DNA (Table 1). Finally, we demonstrate how the genetic data obtained from this sequence capture protocol can be used to identify genetic variation both among and within *P. relictum* lineages (Fig. 4 and Table 2).

The positive relationship between parasitemia and sequence capture success has been documented previously for avian haemosporidians of the genus *Haemoproteus* [10]. In their study of natural infections of *Haemoproteus* parasites, Barrow et al. [10] successfully sequenced infections with parasitemia lower than 0.1% (the lowest of our measured parasitemias in the experimental infection). Our results confirm the relationship between sequence capture success and parasitemia for *P. relictum* parasites and suggest that in samples derived from naturally infected wild birds, many targeted genes may not be fully captured by this protocol.

We identified large spikes in depth of coverage in the mapping inside and outside of probed regions (Fig. 3). The regions with spikes were generally repetitive (low complexity) and BLASTed to ribosomal genes which may have come from the host or the parasite. This suggests that in future use of this probe set, repetitive regions should be removed, as they may not be specific enough to map to their intended targets and analysis should only be conducted on regions that were targeted for sequence capture. We have identified probes to remove in Additional file 4: Table S1. Furthermore, visualizing coverage across the genome (Fig. 3, Additional file 1: Figure S1) brings a deeper understanding of the success of sequence capture and should be conducted in all sequence capture experiments to identify potential mapping errors.

Using data from 25 genes sequenced as part of this study, we identified among- and within-lineage genetic variation. Previous work showed that lineages SGS1 and GRW11 shared MSP1 haplotypes and thus may be recombining [24]. SGS1 and GRW11 share haplotypes from other genes as well (here we show a shared RON3 haplotype; Fig. 4). We also show a shared haplotype between SGS1 and the reference sequence and divergence between samples of SGS1 isolated from different host species (Fig. 4). In fact, across 25 genes, there were 11 nucleotide differences between samples 1309 and 735, both SGS1 lineages but isolated from different host species (Table 2). Future studies can use sequence capture to generate data on whether particular host species can cause such divergence within parasite lineages through selection or whether such divergence is the result of genetic drift, recombination with other parasites, or another mechanism (or combination of mechanisms).

The success of the sequence capture protocol described here is expected based on previous work [10, 23], but is nevertheless important for demonstrating that the protocol can be used to continue phylogeographic and phylogenetic studies across different haemosporidian lineages. Previous analyses using a fragment of the MSP1 gene in these *P. relictum* lineages revealed many new insights, including recombination between SGS1 and GRW11 and the biogeographic history of the lineages [24]. Applying sequence capture to these questions will allow for greater resolution and confirmation of the patterns described previously [29]. It will further provide us with more genetic data that can help identify genes under selection as a result of host–pathogen interactions.

## Conclusions

Like other sequence capture protocols, the success of the one we describe here for *P. relictum* depends on parasitemia and does require large up-front expenses for reagents and sequencing [10]. Because of the dependence on parasitemia, this sequence capture protocol may not work for many field-collected samples. Therefore, identification of high-parasitemia samples through microscopy or quantitative PCR is critical for finding samples that can be used in this protocol. A common benefit of sequence capture protocols is the ability to provide greater resolution to phylogeographic and phylogenetic studies of avian haemosporidians by providing data on genetic variation among and within lineages (Fig. 4 and Table 2). The sequence capture protocol described here will allow researchers to obtain novel genomic data from *P. relictum* and related parasites to better understand the diversification and host-parasite associations of this clade of widespread and extreme host generalist lineages.

## Supplementary Information

**Additional file 1: Figure S1.** Sequencing coverage across the *P. relictum* genome. Each graph of the following figure represents the coverage of each non-archived chromosomes (i.e., chromosomes 1–14 and the apicoplast and mitochondrial genomes) from the *P. relictum* genome (chromosome name appears in upper left-hand side of each graph). Each panel within each of the graphs represents one sample with the sample name above the panel (names follow Additional file 5: Table S2). The *y*-axes were capped at 1000 sequenced bp (depth of coverage) to represent the low coverage regions at a more appropriate scale. However, the spikes often extended past 1000 bp. The *x*-axes are measured in kilobase pairs.

**Additional file 2: Figure S2.** Rarefaction curves of number of probed regions intersected by a read by at least 10 bp as a function of number of reads that mapped to any probed region (assigned reads). A rarefaction curve plotted for each sample showing the number of probed regions intersected by a read from the sample by at least 10 bp in relation to the number of reads that mapped to any probed region (assigned reads) for each sample (the “assigned reads” variable counts the same read more than once if it mapped to more than one probed region). Many of the samples reached asymptotes with relatively high number of probed regions sequenced suggesting more sequencing will not lead to large gains in coverage but may contribute to depth of coverage. Three samples of the parasite lineage SGS1 with parasitemia lower than 1% (1455, 1457, and 1458) group separately from the other SGS1 samples and the rarefaction curves suggest that they had lower numbers of probed regions intersected by a read than the other samples at similar levels of sequencing. This suggests that low parasitemia limited sequence capture success and that this limit likely cannot be fully overcome with greater sequencing. Moreover, sample 51242 (lineage GRW4) had the most sequencing of all the samples (> 15 million total reads; Additional file 5: Table S2), but still had a relatively low number of probed regions intersected by a read and relatively few assigned reads. Sample 51242 also had low parasitemia (< 1%) and represented the most divergent of the lineages (GRW4).

**Additional file 3: File S1.** Sequence capture probe set. All 120-bp probes used for sequence capture in this study.

**Additional file 4: Table S1.** Sequence capture targets. List of sequence capture probes, the *P. relictum* chromosome, start and end coordinates, and target gene, description of target gene that the probes targeted, and a column titled “reason_to_filter”. The reason_to_filter column indicates reasons (e.g., repetitive region, spikes in coverage) to remove particular probes from future designs. The probes that did not map to the version 54 of the reference genome are indicated in the target gene description column.

**Additional file 5: Table S2.** Sample metadata and sequence results. List of samples used in this study, their names, the cyt *b* lineages they represent, where and when and from what host species they were isolated and the host species they were sequenced from, additional data including parasitemia and sequencing data are also included.

**Additional file 6: Table S3.** List of 25 genes selected for haplotype network analysis and concatenated distance matrix including their coordinates in the genome and aligned length (bp).

**Additional file 7: Table S4.** Number of reads mapping to each targeted gene (Geneid) by at least 10 bp for each sample.

**Additional file 8: Table S5.** Number of reads mapping to each probe (Probeid) by at least 10 bp for each sample. Probes with no mapping are not presented in the table.

## Data Availability

Raw sequence reads generated in this study have been deposited in the EMBL-EBI European Nucleic Acid repository under accession ID: PRJEB48854.

## Abbreviations

BLAST: Basic Local Alignment Search Tool
bp: Base pair
cyt *b*: Cytochrome b
DNA: Deoxyribonucleic acid
Mbp: Megabase pair
MSP1: Merozoite surface protein 1
MSP8: Merozoite surface protein 8
PCR: Polymerase chain reaction
RFC1: Replication factor C subunit 1
RON3: Rhoptry neck protein 3
RON12: Rhoptry neck protein 12
RNA: Ribonucleic acid
