# Supplementary material for: Genomic sequence capture of Plasmodium relictum in experimentally infected birds
Source: Parasit Vectors. 2022 Jul 29;15:267. doi: 10.1186/s13071-022-05373-w (PMC9336033; doi:10.1186/s13071-022-05373-w)

**Additional file 1: Figure S1.** Each graph of the following figure represents the coverage of each non-archived chromosomes (i.e., chromosomes 1 – 14 and the apicomplast and mitochondrial genomes) from the *P. relictum* genome (chromosome name appears in upper left-hand side of each graph). Each panel within each of the graphs represents one sample with the sample name above the panel (names follow Supplementary Table 2). The y-axes were capped at 1,000 sequenced bp (depth of coverage) to represent the low coverage regions at a more appropriate scale. However, the spikes often extended past 1,000 bp. The x-axes are measured in kilobase pairs.


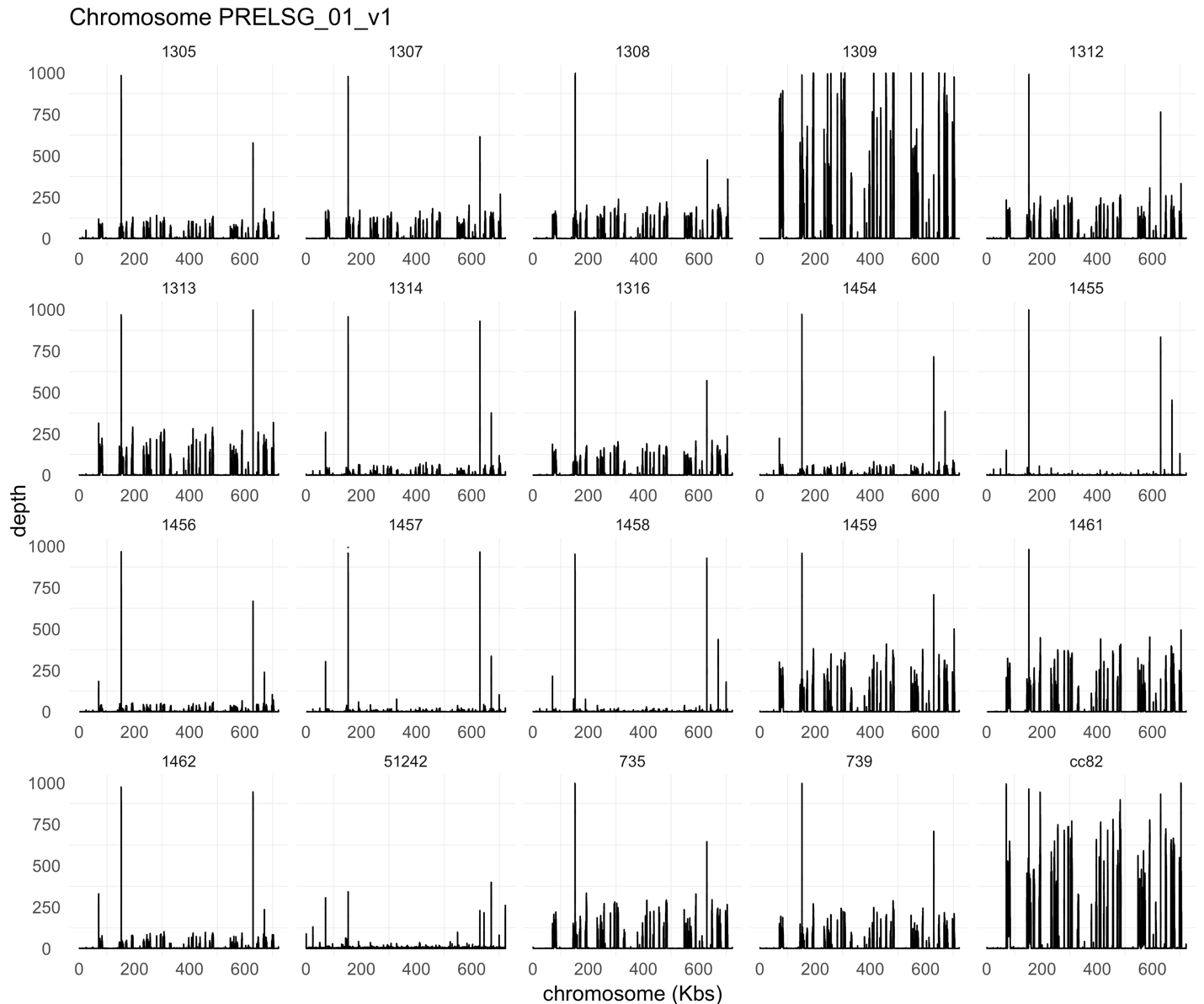

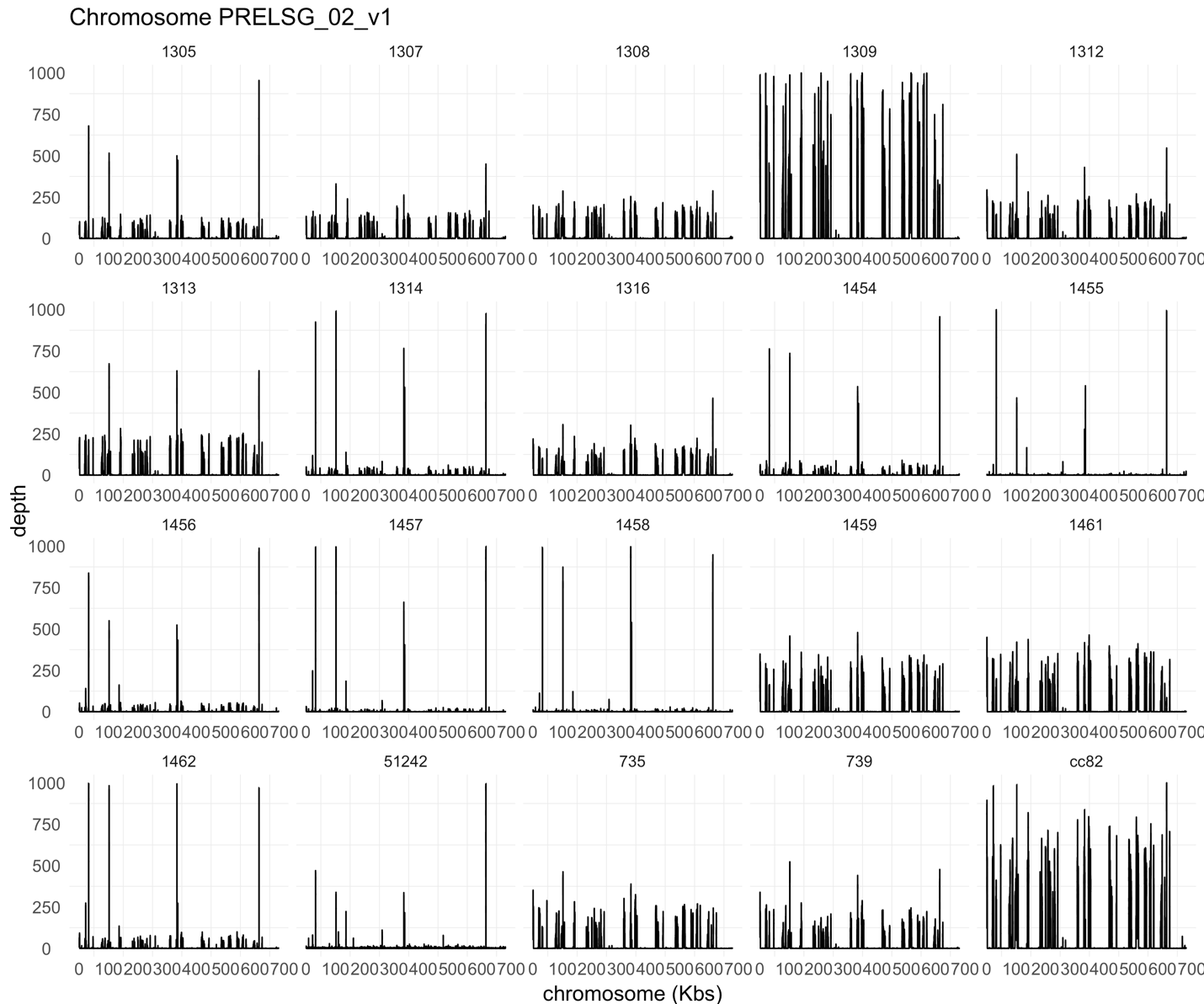

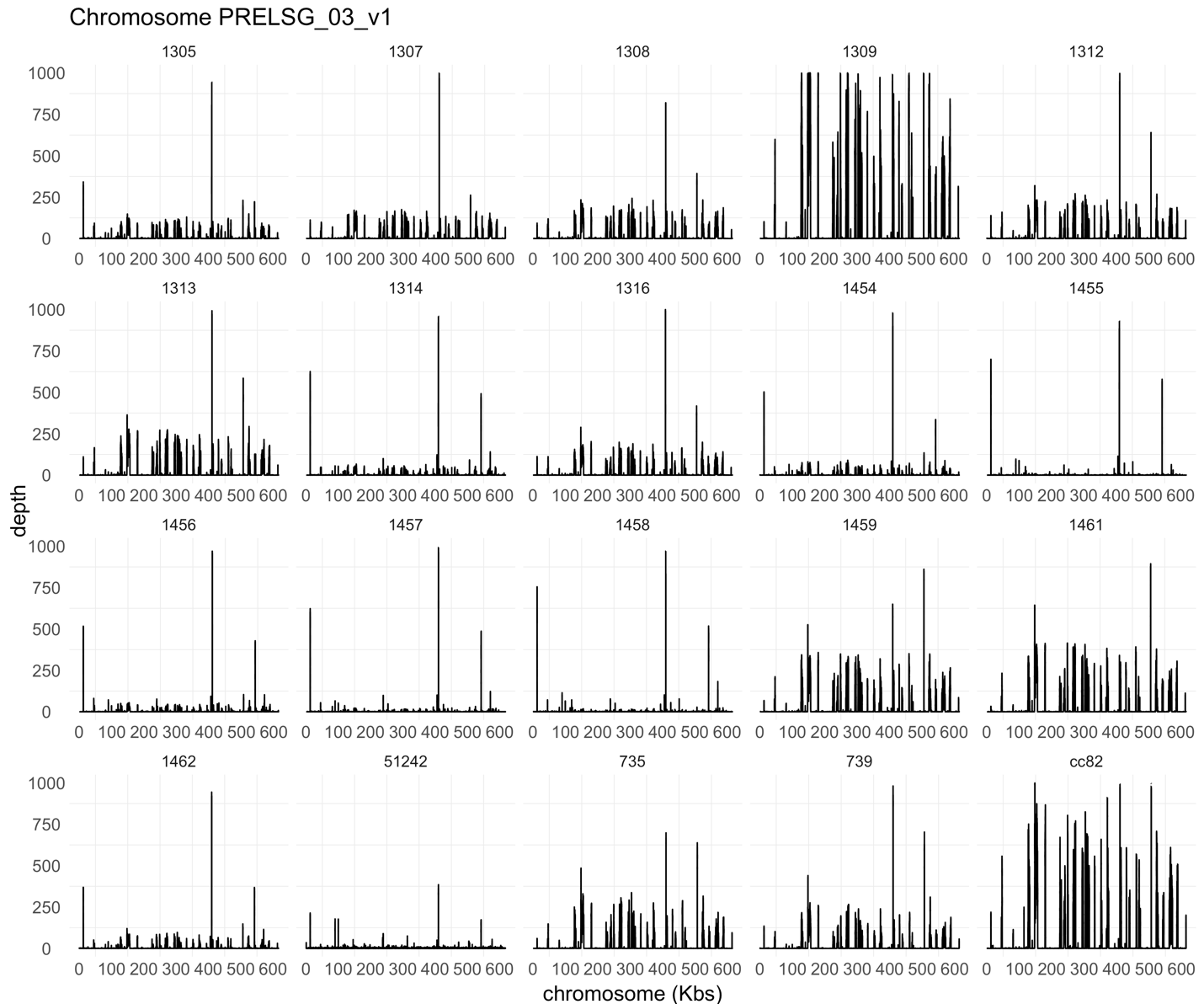

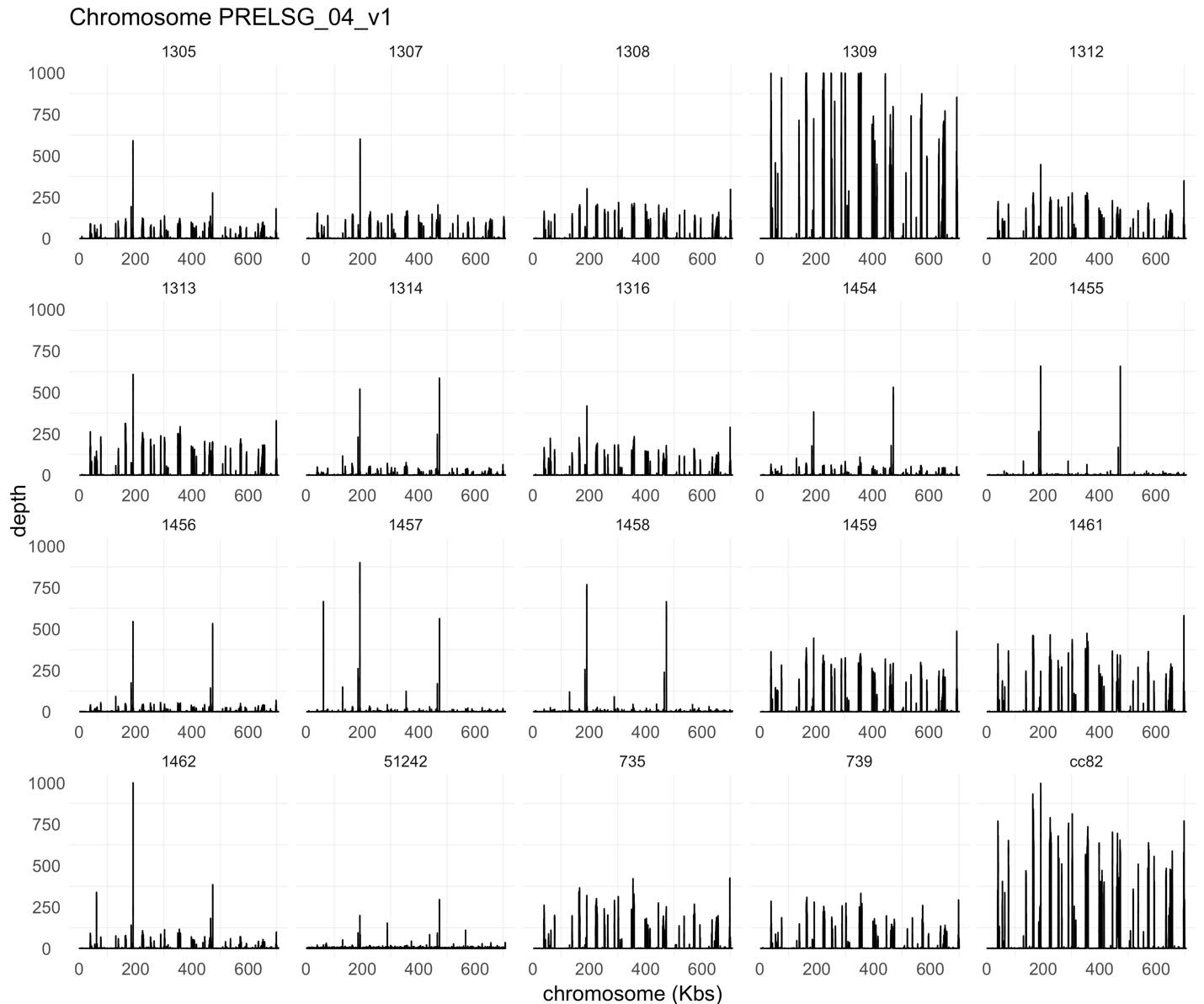

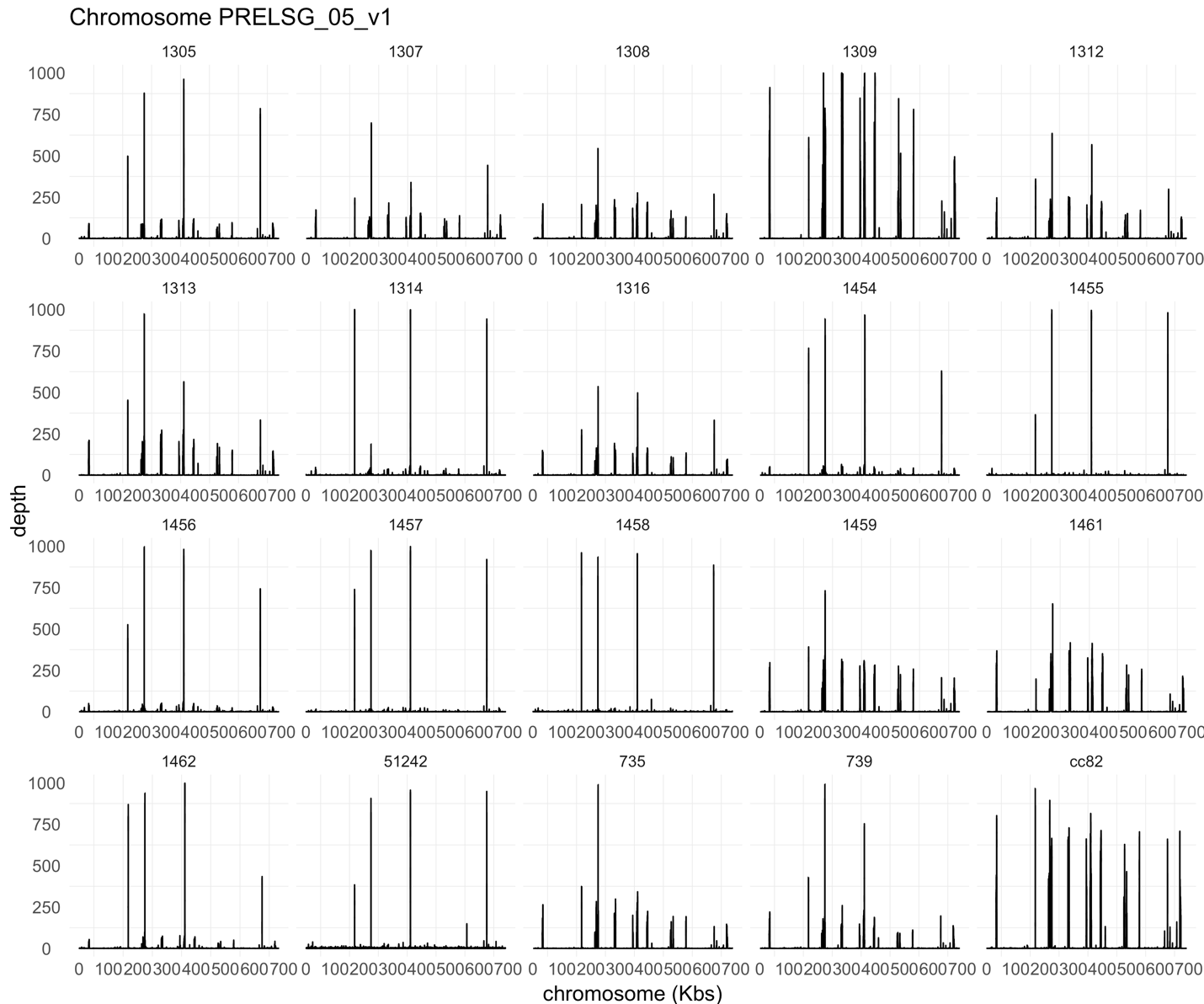

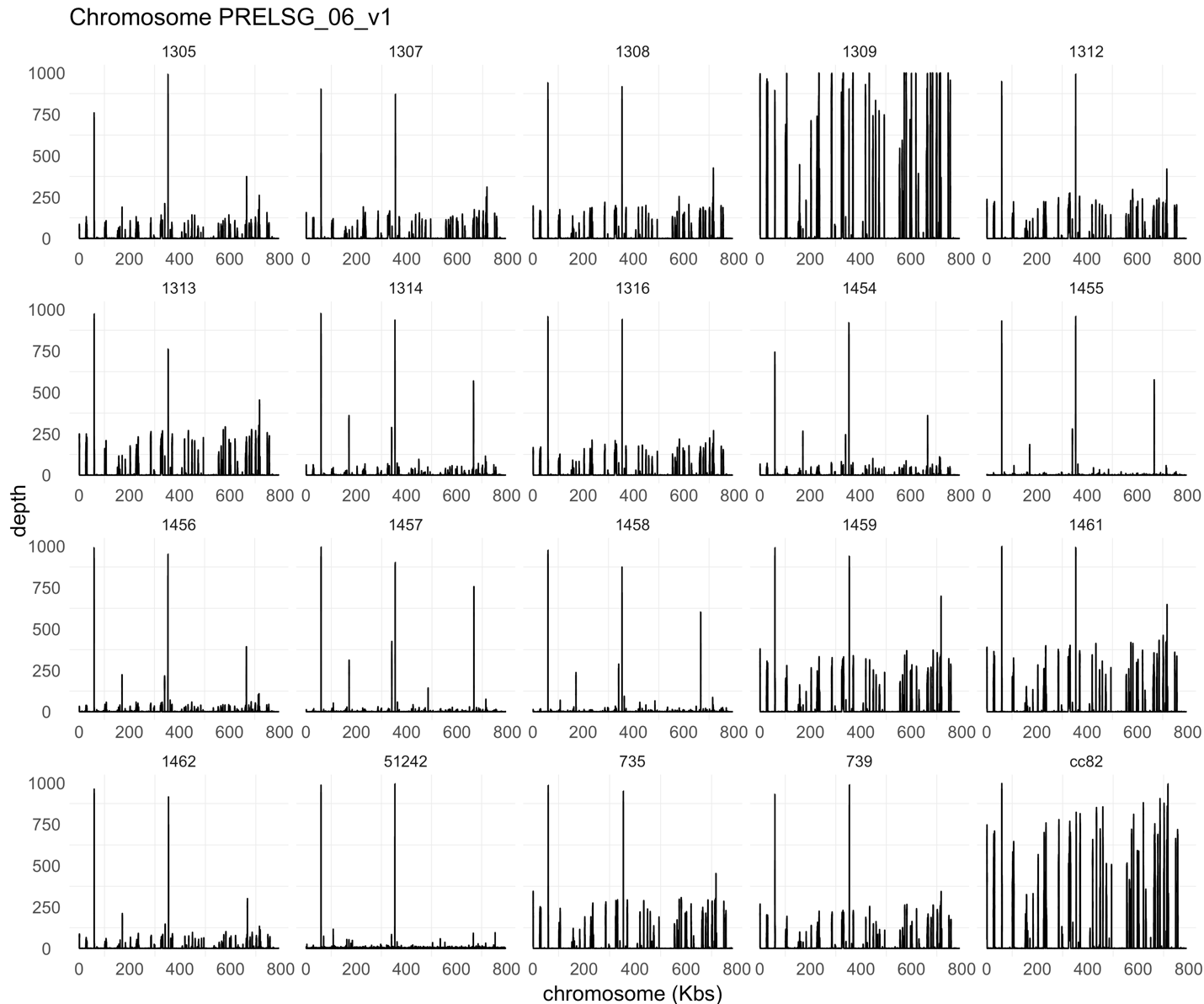

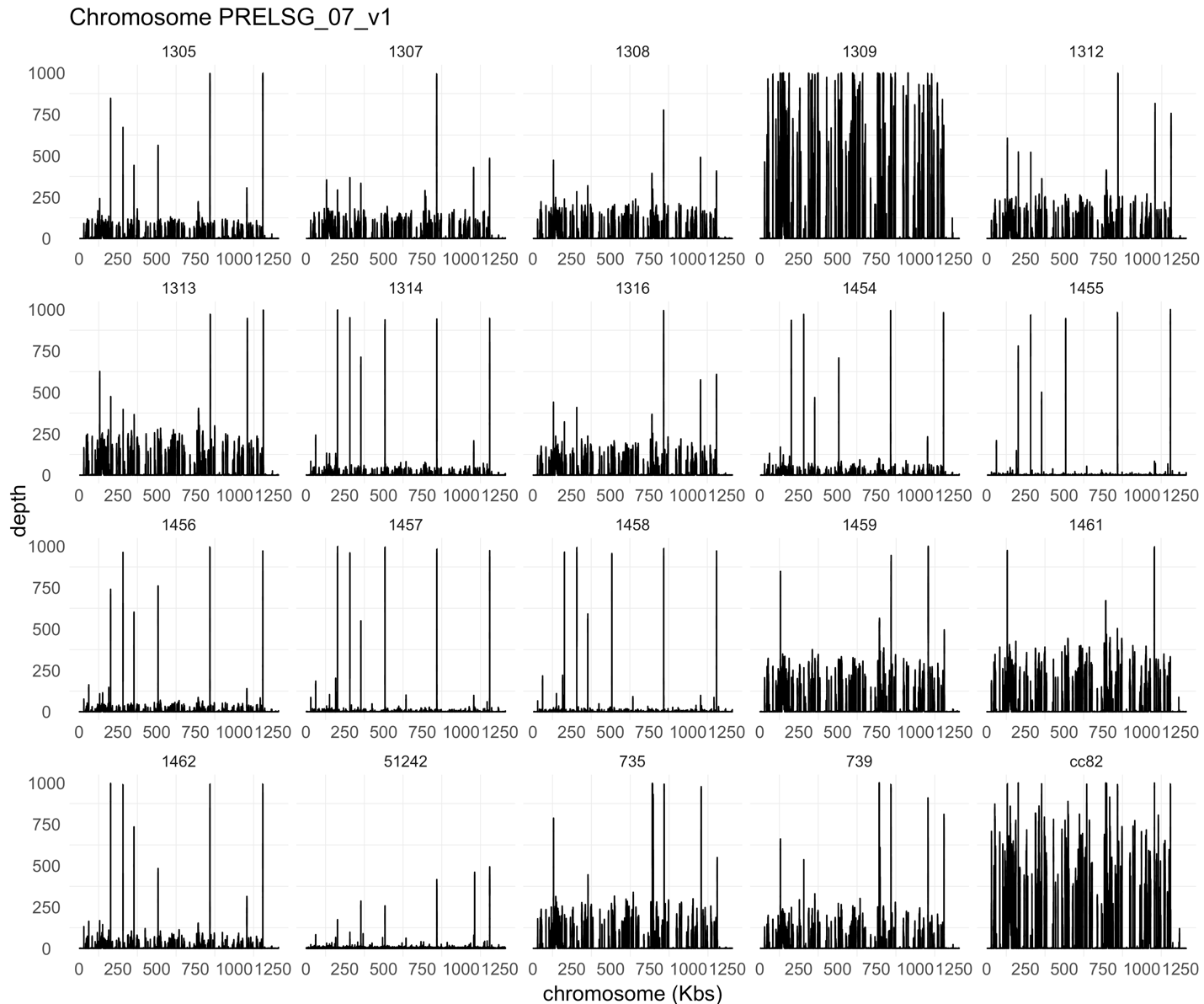

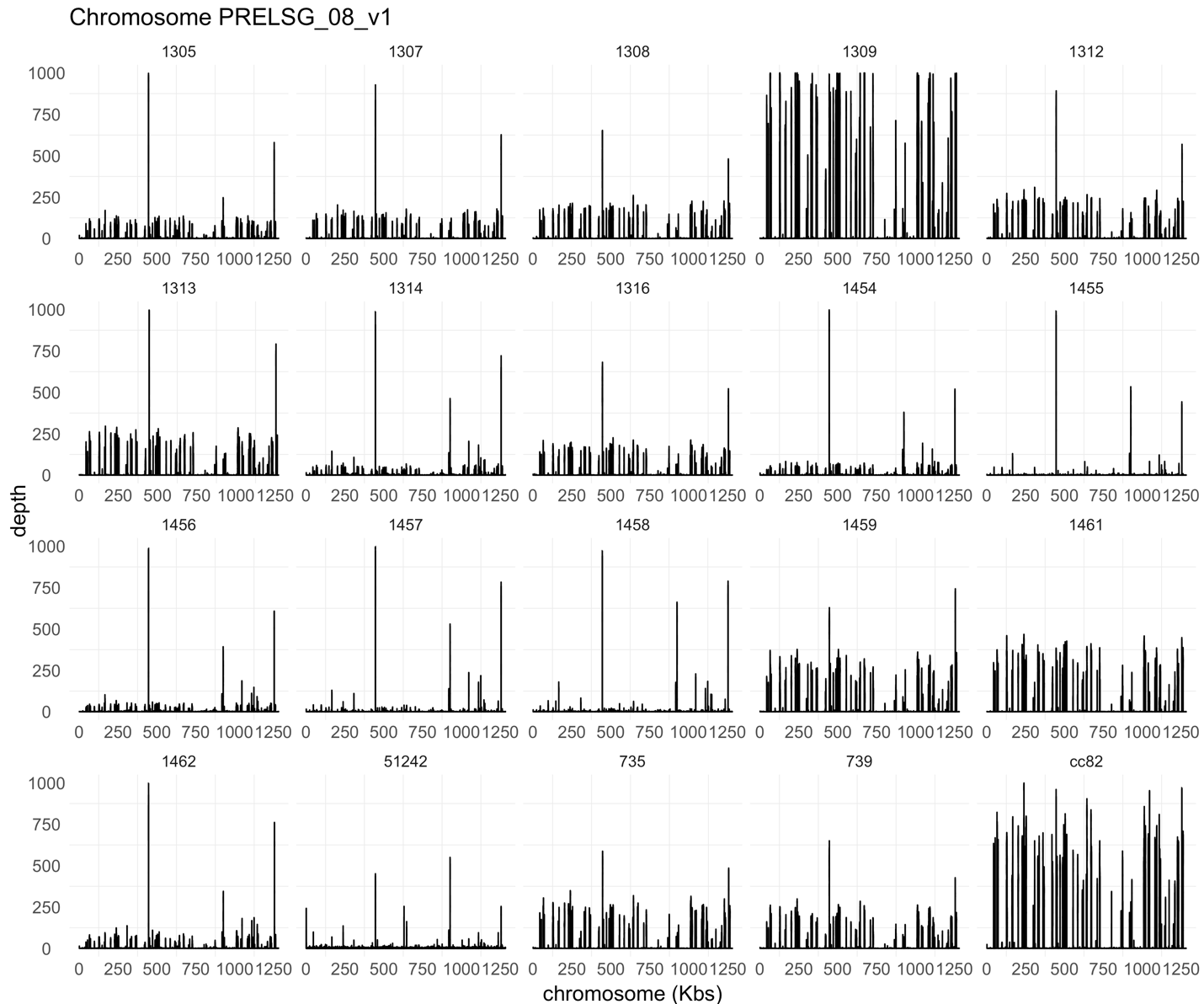

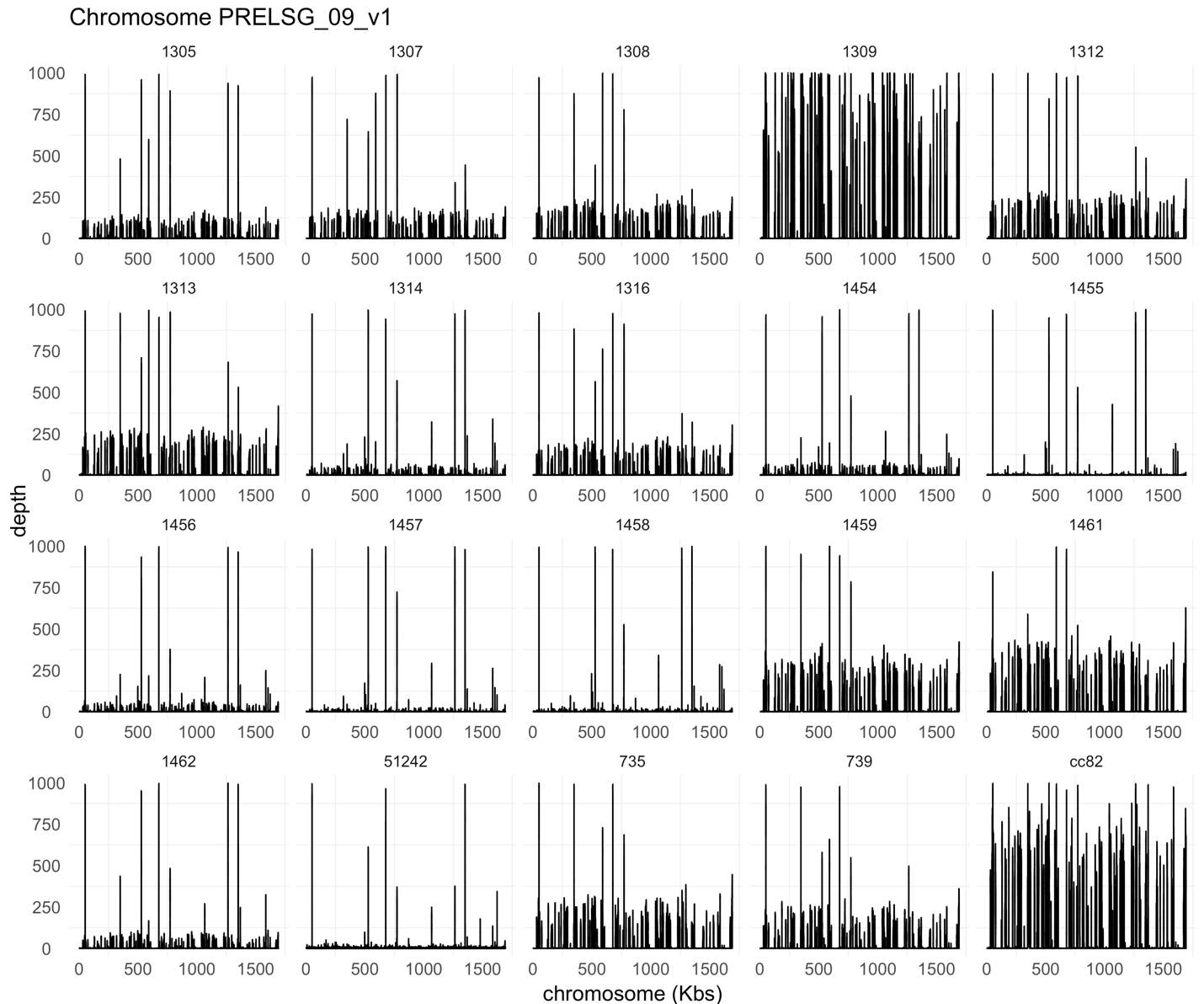

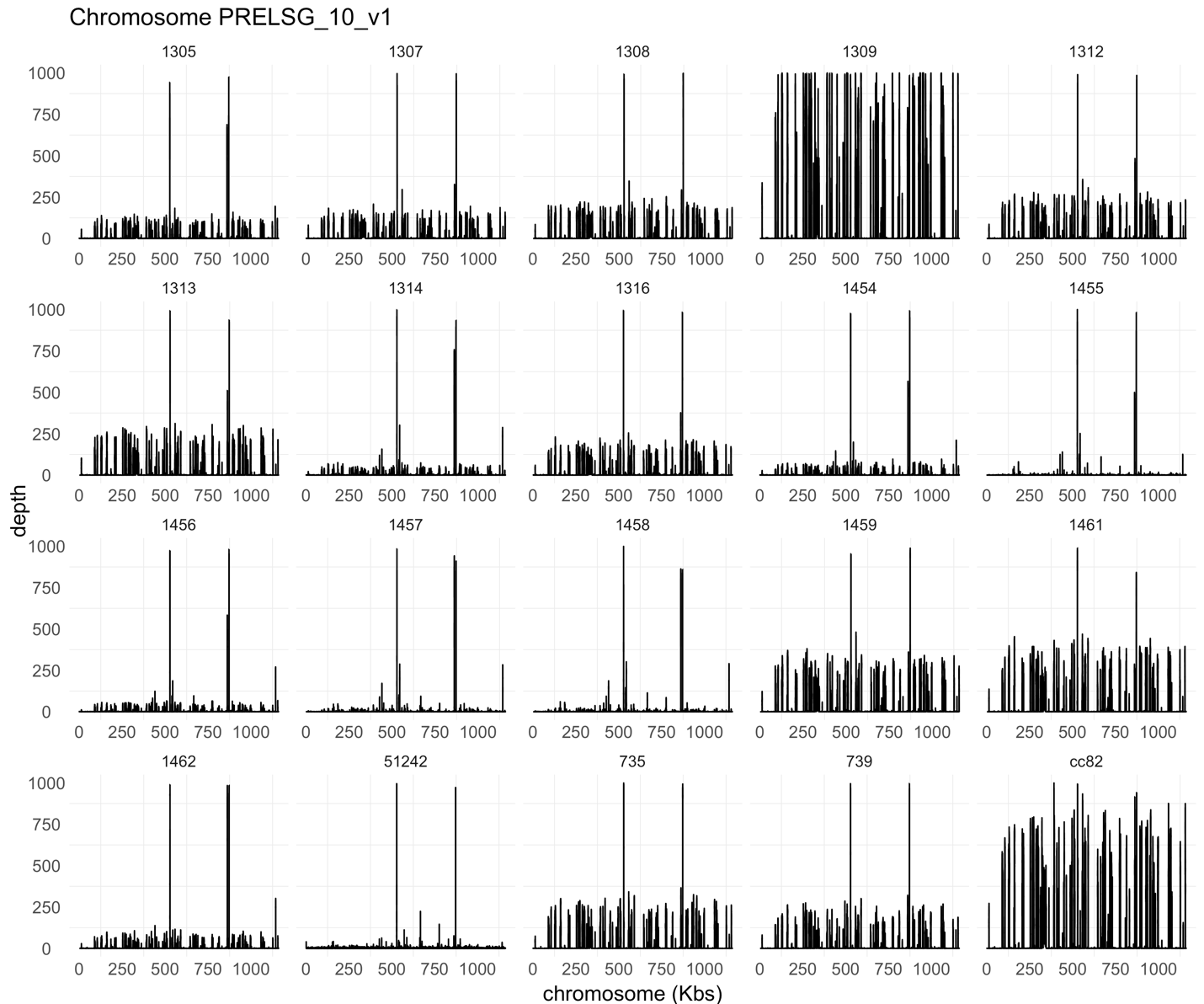

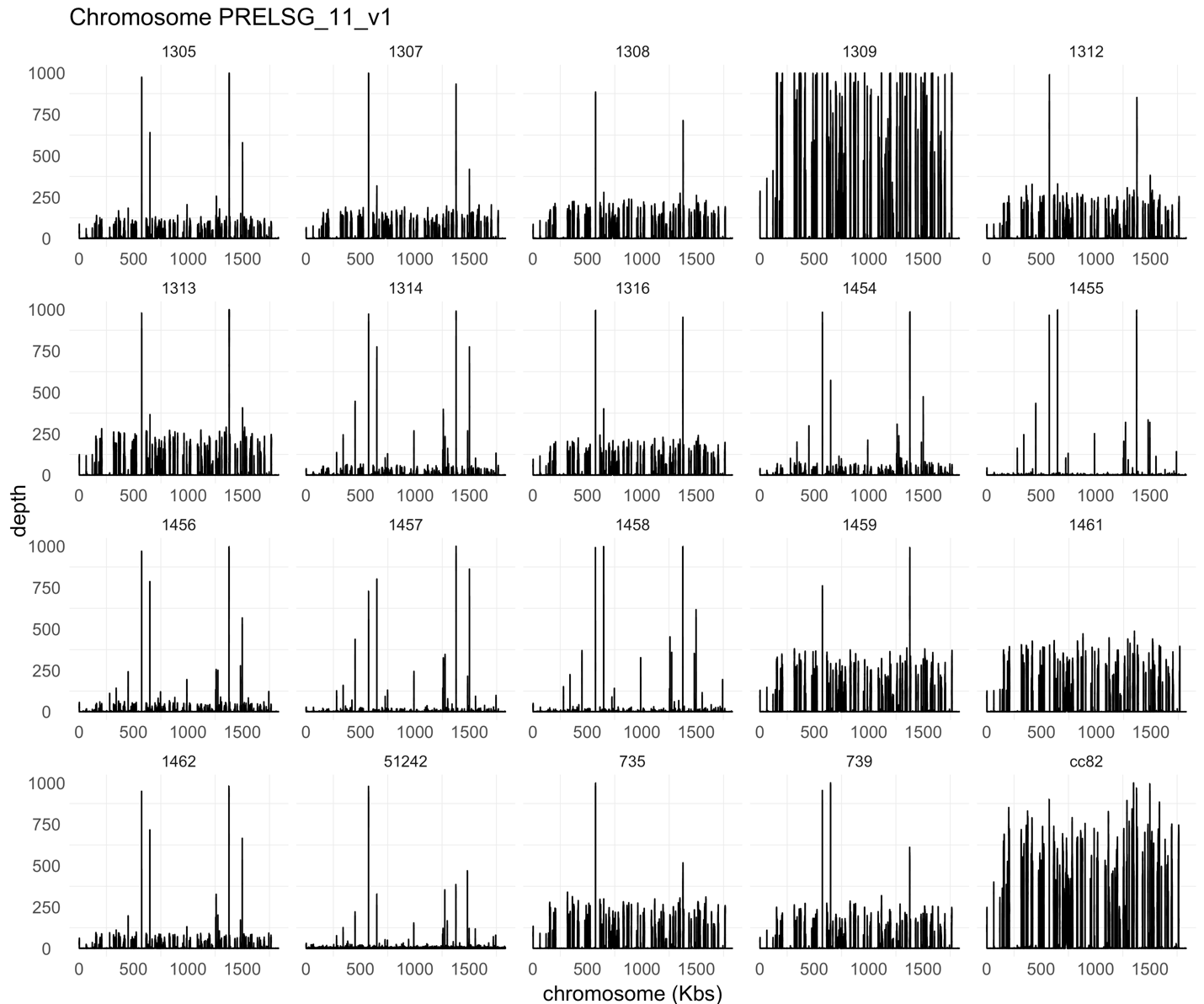

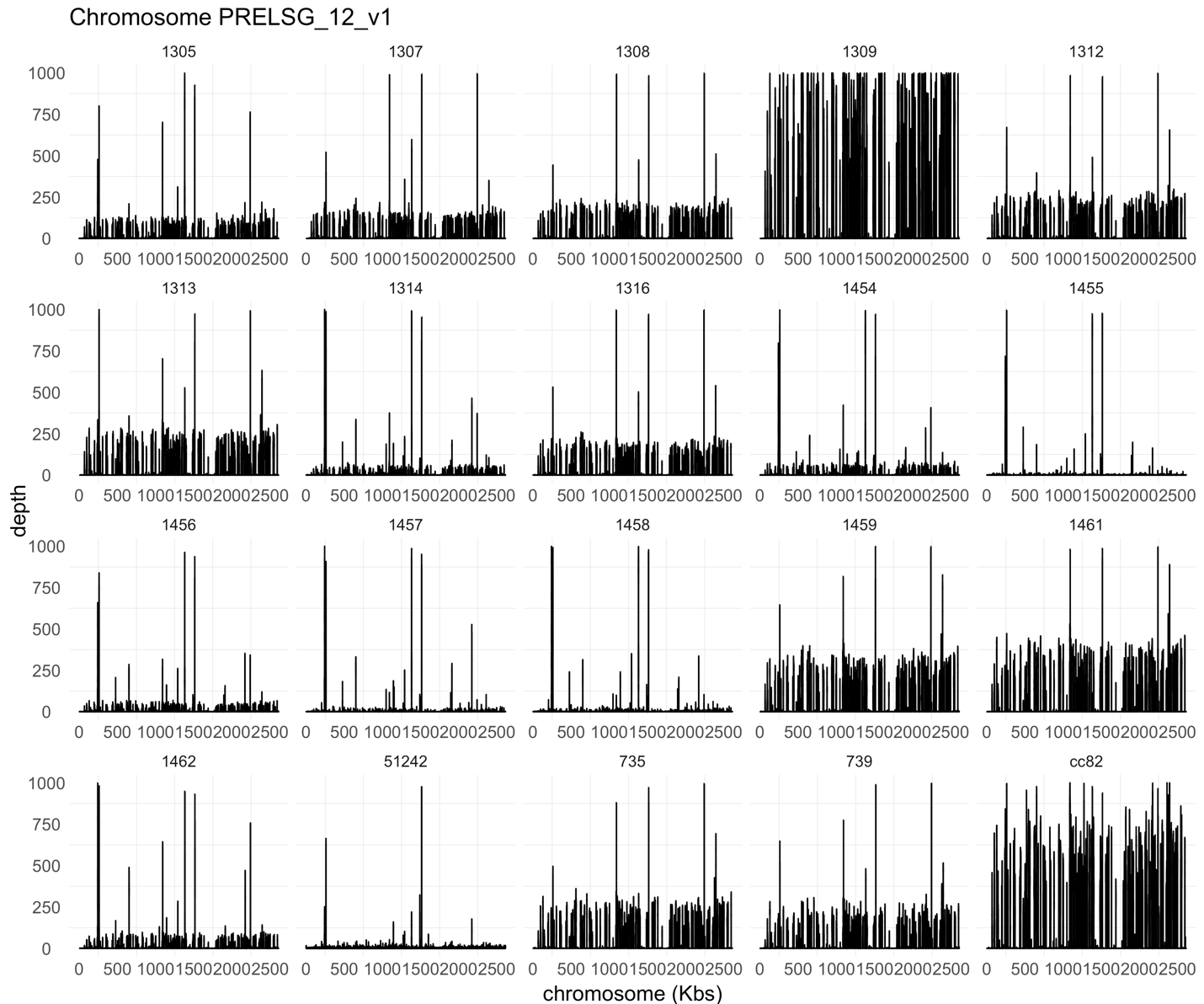

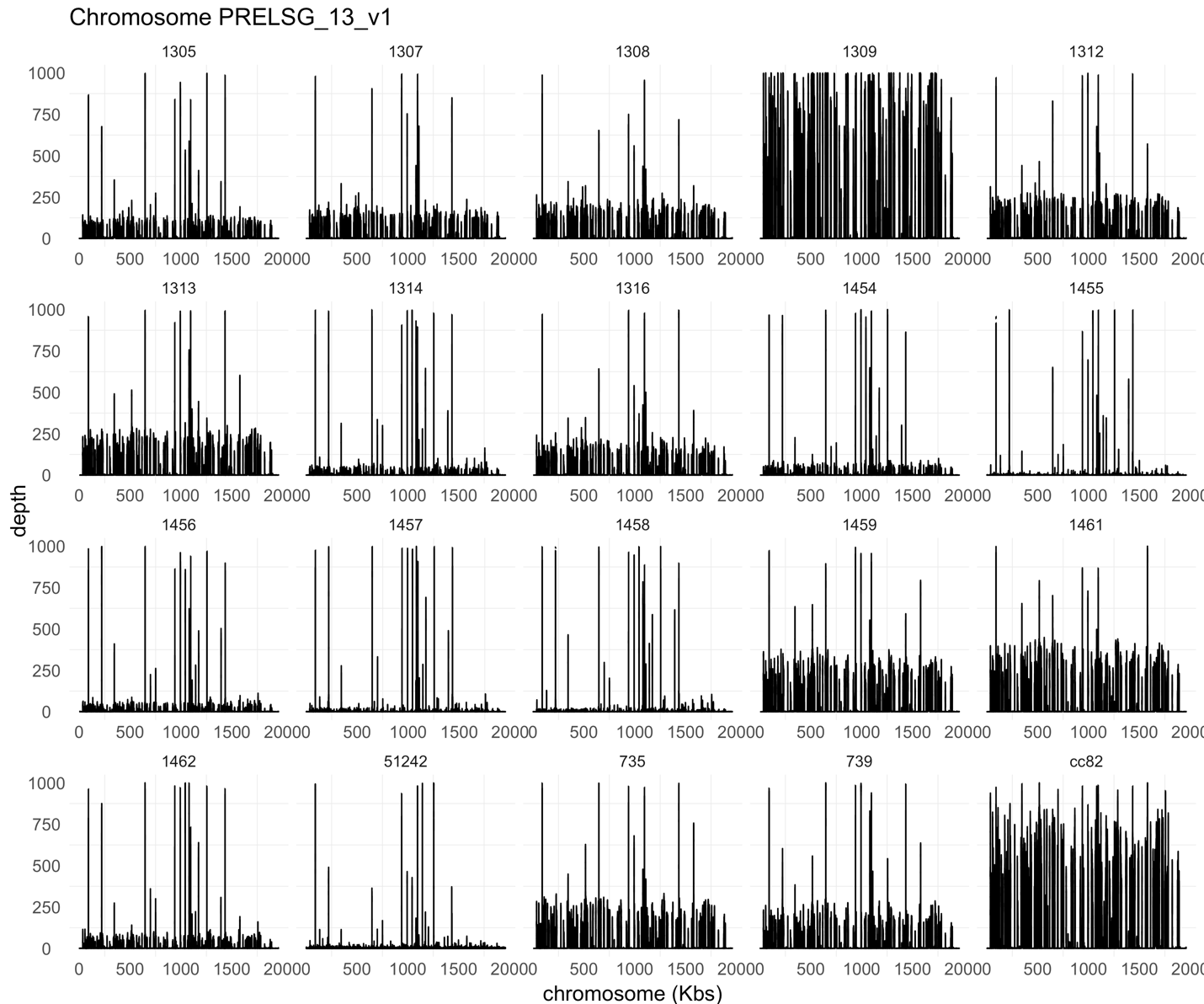

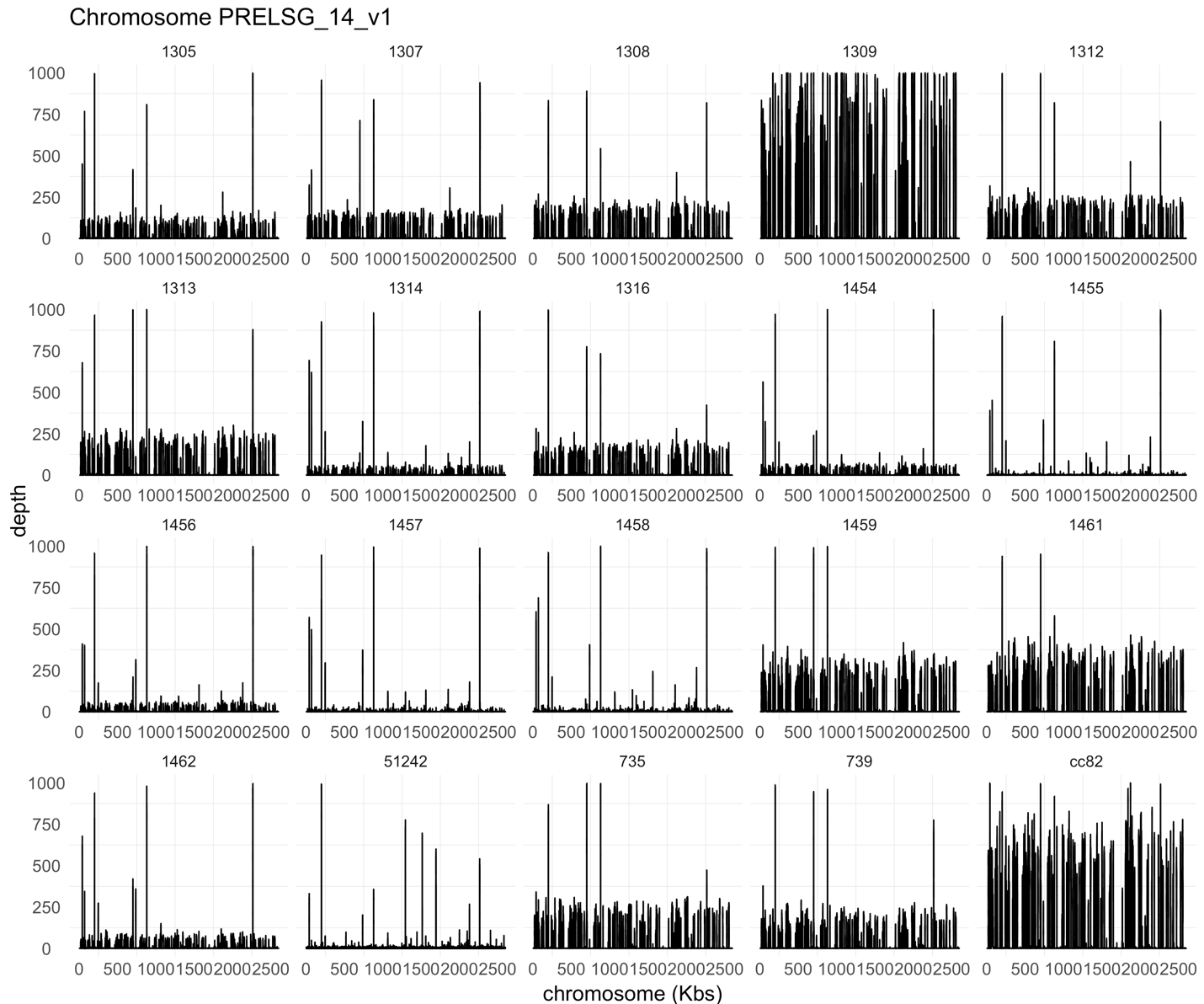

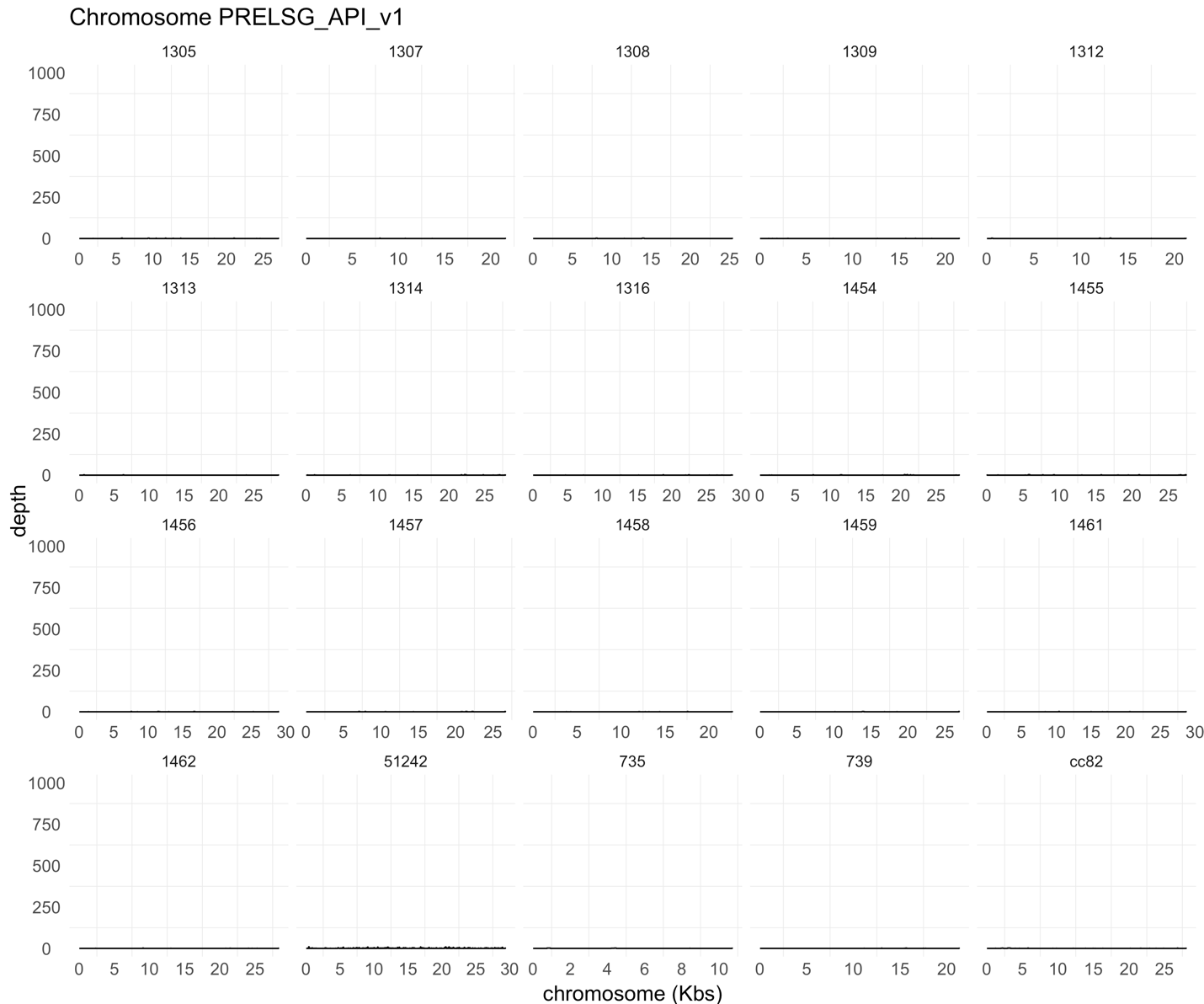

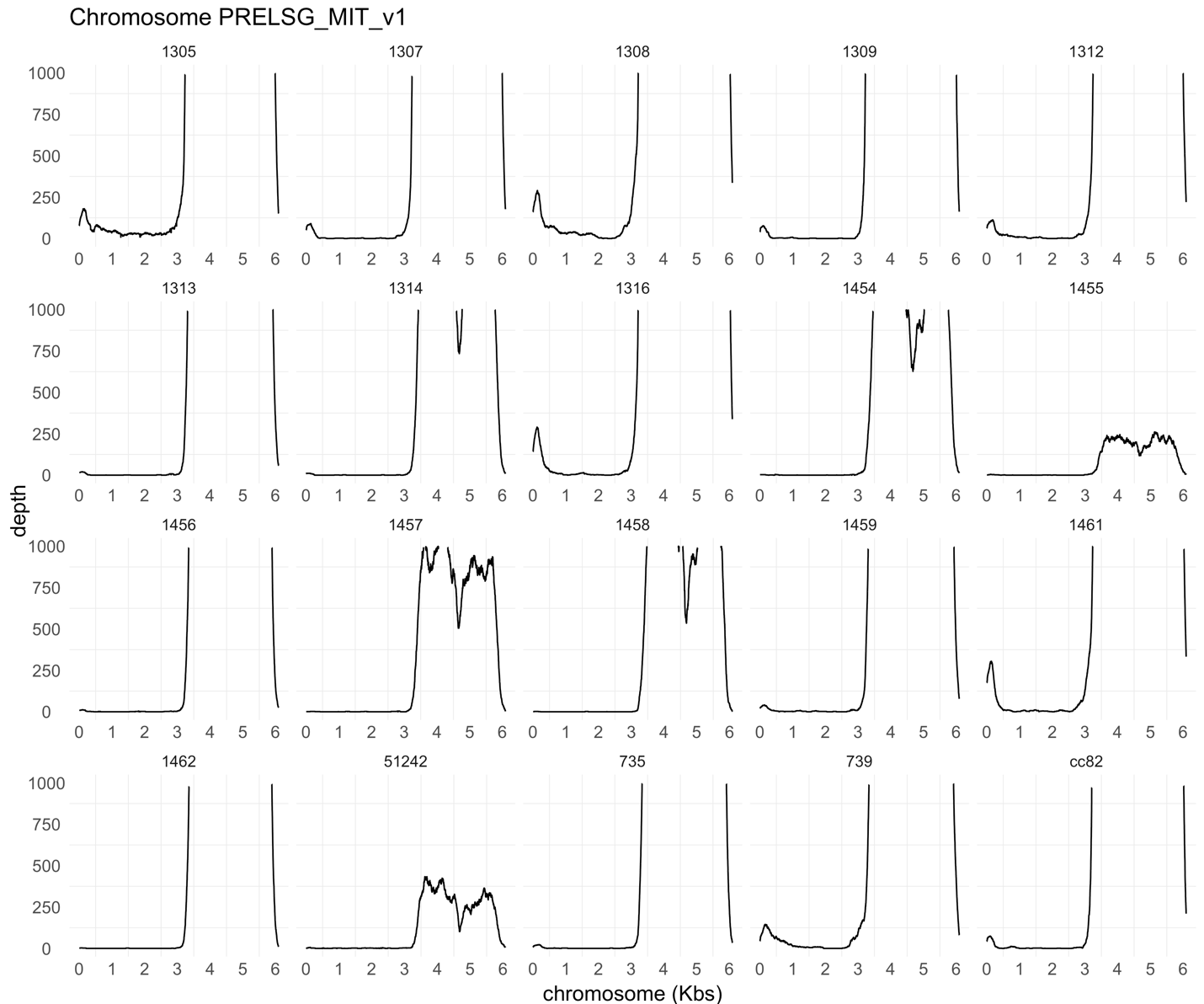

Supplement: Supplementary file 1 — Additional file 1: Figure S1. Sequencing coverage across the P. relictum genome. Each graph of the following figure represents the coverage of each non-archived chromosomes (i.e., chromosomes 1–14 and the apicoplast and mitochondrial genomes) from the P. relictum genome (chromosome name appears in upper left-hand side of each graph). Each panel within each of the graphs represents one sample with the sample name above the panel (names follow Additional file 5: Table S2). The y-axes were capped at 1000 sequenced bp (depth of coverage) to represent the low coverage regions at a more appropriate scale. However, the spikes often extended past 1000 bp. The x-axes are measured in kilobase pairs. [file 13071_2022_5373_MOESM1_ESM.docx]
