# Supplementary figures and images for: Genomic sequence capture of Plasmodium relictum in experimentally infected birds
Source: Parasit Vectors. 2022 Jul 29;15:267. doi: 10.1186/s13071-022-05373-w (PMC9336033; doi:10.1186/s13071-022-05373-w)

## rarefaction curves for all samples

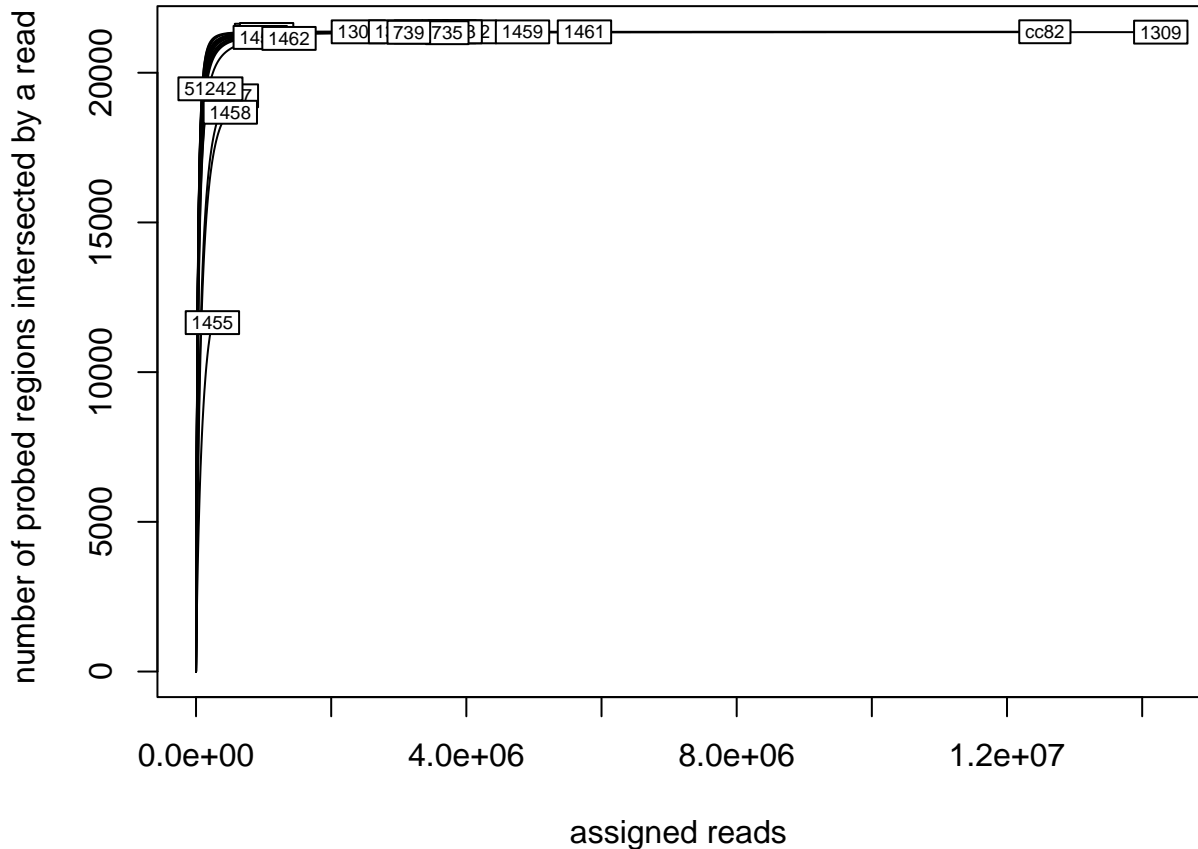

Supplement: Supplementary file 2 — Additional file 2: Figure S2. Rarefaction curves of number of probed regions intersected by a read by at least 10 bp as a function of number of reads that mapped to any probed region (assigned reads). A rarefaction curve plotted for each sample showing the number of probed regions intersected by a read from the sample by at least 10 bp in relation to the number of reads that mapped to any probed region (assigned reads) for each sample (the “assigned reads” variable counts the same read more than once if it mapped to more than one probed region). Many of the samples reached asymptotes with relatively high number of probed regions sequenced suggesting more sequencing will not lead to large gains in coverage but may contribute to depth of coverage. Three samples of the parasite lineage SGS1 with parasitemia lower than 1% (1455, 1457, and 1458) group separately from the other SGS1 samples and the rarefaction curves suggest that they had lower numbers of probed regions intersected by a read than the other samples at similar levels of sequencing. This suggests that low parasitemia limited sequence capture success and that this limit likely cannot be fully overcome with greater sequencing. Moreover, sample 51242 (lineage GRW4) had the most sequencing of all the samples (> 15 million total reads; Additional file 5: Table S2), but still had a relatively low number of probed regions intersected by a read and relatively few assigned reads. Sample 51242 also had low parasitemia (< 1%) and represented the most divergent of the lineages (GRW4). [file 13071_2022_5373_MOESM2_ESM.pdf]
